# Dinuclear Gallium(III) Complex With 1,3-Propanediamine-*N,N′*-Diacetate: Structural Characterization, Antimicrobial Activity, and DNA/BSA Interactions

**DOI:** 10.1155/bca/8097589

**Published:** 2025-04-12

**Authors:** Bojana V. Pantović, Darko P. Ašanin, Žiko Milanović, Franc Perdih, Tatjana Ilic-Tomic, Dušanka D. Radanović, Iztok Turel, Miloš I. Djuran, Biljana Đ. Glišić

**Affiliations:** ^1^Department of Chemistry, Faculty of Science, University of Kragujevac, Radoja Domanovića 12, Kragujevac 34000, Serbia; ^2^Department of Science, Institute for Information Technologies Kragujevac, University of Kragujevac, Jovana Cvijića bb, Kragujevac 34000, Serbia; ^3^Faculty of Chemistry and Chemical Technology, University of Ljubljana, Večna pot 113, Ljubljana SI-1000, Slovenia; ^4^Institute of Molecular Genetics and Genetic Engineering, University of Belgrade, Vojvode Stepe 444a, Belgrade 11042, Serbia; ^5^Institute of Chemistry, Technology and Metallurgy, University of Belgrade, Njegoševa 12, Belgrade 11000, Serbia; ^6^Department of Chemical and Biological Sciences, Serbian Academy of Sciences and Arts, Knez Mihailova 35, Belgrade 11000, Serbia

**Keywords:** aminocarboxylate ligand, antimicrobial activity, DNA/BSA interactions, gallium(III) complexes, structural characterization

## Abstract

In this study, a tetradentate 1,3-propanediamine-*N,N′-*diacetate (1,3-pdda^2−^) was utilized for the synthesis of a dinuclear gallium(III) complex, uns-*cis*-[Ga(1,3-pdda)(*µ*-OH)]_2_^.^2H_2_O (**1**). Complex **1** was characterized using IR and NMR (^1^H and ^13^C) spectroscopy, and its crystal structure was determined by single-crystal X-ray diffraction analysis. Both Ga(III) ions in Complex **1** exhibit octahedral geometry, with each ion coordinated by two nitrogen and two oxygen atoms from the 1,3-pdda^2–^ ligand, as well as two oxygen atoms from the bridging hydroxyl groups. IR and NMR (^1^H and ^13^C) spectra were simulated using DFT methods, showing a high degree of correlation with experimental data. Hirshfeld surface analysis provided insights into intermolecular interactions, with H⋯O and H⋯H interactions contributing significantly to the crystal stability. The antimicrobial potential of Complex **1** was evaluated alongside previously synthesized gallium(III) complexes, Na[Ga(1,3-pdta)]·3H_2_O (**2**) and Ba[Ga(1,3-pndta)]_2_·3H_2_O (**3**), with 1,3-pdta^4−^ (1,3-propanediamine-*N,N,N′,N′*-tetraacetate) and 1,3-pndta^4−^ ((±)-1,3-pentanediamine-*N,N,N′,N′*-tetraacetate), respectively. Among all the tested microbial species, the gallium(III) complexes have shown selective activity against *Pseudomonas aeruginosa* PAO1 strain and were able to reduce pyocyanin production by 40–43% in the clinical isolate BK25H of this bacterium. Moreover, Complexes **1**–**3** can modulate the quinolone-mediated quorum sensing system in *P. aeruginosa* PAO1. Interaction studies with calf thymus DNA (ct-DNA) and bovine serum albumin (BSA) were conducted to evaluate the binding affinity and mode of interaction of Complex **1** with key biomolecules, aiming to assess its potential for transport via serum proteins and its safety profile in terms of DNA interactions. Spectrofluorimetric experiments and molecular docking revealed that Complex **1** binds strongly to the Site I on BSA, with weaker interactions at the Site II. While spectrofluorimetric studies showed that Complex **1** has a slight affinity for minor groove binding or intercalation to ct-DNA, docking studies suggested some minor groove binding, especially in larger DNA sequences, with enhanced stabilization in 10-bp-DNA through hydrogen and carbon bonds.

## 1. Introduction

The ethylenediamine-*N,N′*-diacetate anion (edda^2−^), containing two amine nitrogen and two carboxylate oxygen donor atoms, is one of the most used aminocarboxylate ligands for the synthesis of transition metal complexes. The tetradentate coordination of this ligand (N_2_O_2_ coordination) results in the formation of three chelate five-membered rings, one ethylenediamine, and two acetate rings. While the ethylenediamine backbone ring is located at the equatorial plane with exclusively *cis*-position of the coordinated nitrogen atoms, the other two five-membered acetate rings can have an axial or equatorial arrangement in the octahedral geometry (Figure S1). It was found that factors which determine the distribution of geometrical isomers and structural properties of octahedral metal complexes with edda^2˗^ and structurally related ligands include the *d*-electron configuration, the size of the central metal ion, and the size of the diamine chain of N_2_O_2_ aminocarboxylate type of ligands [1, 2]. The coordination reactions of edda^2˗^ ligand with various transition metal ions have been extensively investigated [1–9]. In the case of octahedral cobalt(II/III) [1, 3–6], chromium(III) [7, 8], nickel(II) [6], zinc(II) [6], and copper(II) [9] complexes, the tetradentate coordination of this ligand with five-membered acetate rings in axial position (symmetrical-*cis* or s-*cis* configuration; Figure S1) occurred.

Concerning edda^2−^, structurally similar 1,3-pdda^2−^ (1,3-propanediamine-*N,N′*-diacetate) and eddp^2−^ (ethylenediamine-*N,N′*-di-3-propionate) ligands with longer diamine or carboxylate chains, respectively, upon their tetradentate coordination, preferably form mixed ligand octahedral complexes of uns-*cis* geometry (unsymmetrical-*cis* coordination with five-membered acetate or six-membered propionate rings in equatorial and axial positions; Figure S1) with different transition metal ions, such as cobalt(III) [10–15], chromium(III) [16, 17], and nickel(II) [18].

In recent years, gallium and its compounds have been used and investigated for numerous medical applications, especially in the treatment of cancer, metabolic bone diseases, and various infectious diseases [19, 20]. Gallium(III) ion shares similarities with iron(III) in terms of ionic radius and chemical properties, such as electronegativity, electron affinity, and coordination number [21]. In contrast to iron ions with different oxidation states, gallium(III) ion is redox-inactive under physiological conditions. Since iron(III) is crucial for bacterial growth and maintenance, gallium(III) can act as an iron(III) mimic and can infiltrate into the bacterial system. As a result, gallium(III) is often mistakenly identified and thus further destroys an iron(III) metabolism by binding with a series of iron-binding proteins [22] or many enzymes [23], providing remarkable opportunities in medical applications. An approach that is based on gallium–iron similarities is sometimes called also “Trojan Horse” strategy [24].

Different aminocarboxylate types of ligands are shown as good chelating agents for gallium(III) ion forming very stable complexes in water solution [25–35]. In most cases, the ligands used for the synthesis of gallium(III) complexes contain two amine nitrogen atoms and two or four carboxylate oxygen donor atoms (N_2_O_2_^−^ or N_2_O_4_ type of ligands) [25–35]. The crystal structures of different octahedral [Ga(edda)(L)] types of complexes (L is bidentate amino acid (NO coordinated), such as glycine, (L)-asparagine, (L)-proline, (L)-threonine, and (L)-leucine) confirmed tetradentate N_2_O_2_ coordination of edda^2−^ ligand with both five-membered acetate rings in axial positions (s-*cis* coordination) [35]. The same s-*cis* coordination of edda^2−^ ligand was also observed for two hydroxide-bridged gallium(III) complexes, [Ga(edda)(*μ*-OH)]_6_^.^xH_2_O and [Ga(edda)(*μ*-OH)]_2_ [35]. In these complexes, 2 Ga(edda)^+^ units are connected by two neighboring hydroxide ligands, forming the ring-like structure. The octahedral coordination spheres of the GaN_2_O_4_ surroundings in both complexes are slightly distorted, which can be observed from interatomic distances and angles between gallium(III) and the nitrogen and oxygen atoms of the ligands [35].

**Figure 1:**
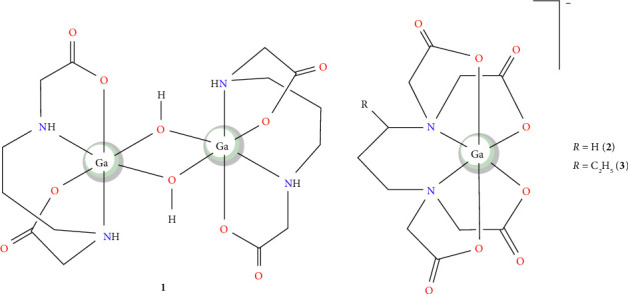
Structural formula of gallium(III) complexes with 1,3-pdda^2−^ (**1**), 1,3-pdta^4−^ (**2**), and 1,3-pndta^4−^ (**3**) ligands used for antimicrobial evaluation.

In the present study, the coordination of 1,3-pdda^2−^ ligand with gallium(III) ion was investigated. The main goal of this study was to address how the prolongation of the diamine chain with one methylene group in this ligand influences its coordination ability and distribution of geometrical isomers of the corresponding 1,3-pdda-gallium(III) complexes with respect to the analog edda^2−^ complexes. The synthesis, spectroscopic, and crystallographic characterization of a new dinuclear gallium(III) complex, uns-*cis*-[Ga(1,3-pdda)(*µ*-OH)]_2_^.^2H_2_O (**1**), was reported (Figure 1). The Hirshfeld surface analysis (HSA) provided insights into the intermolecular interactions within the crystal structure. Theoretical simulations of IR and NMR (^1^H and ^13^C) spectra were performed and compared with experimental data to support the structure of Complex **1**. The antimicrobial activity of Complex **1** and two gallium(III) complexes recently reported, Na[Ga(1,3-pdta)]·3H_2_O (**2**) (1,3-pdta^4−^ = 1,3-propanediamine-*N,N,N′,N′*-tetraacetate) and Ba[Ga(1,3-pndta)]_2_·3H_2_O (**3**) (1,3-pndta^4−^ = (±)-1,3-pentanediamine-*N,N,N′,N′*-tetraacetate) (Figure 1) [34], was evaluated against different bacterial and *Candida* strains, while their cytotoxic effects were tested on the healthy human fibroblasts MRC-5. Additionally, fluorescence emission spectroscopy and molecular docking studies were conducted to explore the binding interactions of dinuclear gallium(III) Complex **1**, particularly focusing on its affinity for calf thymus DNA (ct-DNA) and bovine serum albumin (BSA).

## 2. Materials and Methods

### 2.1. Chemicals and Instrumentations

All commercially reagent-grade chemicals were used without further purification. Constituents of bacteriological media were molecular biology reagent grade. Peptone and tryptone were purchased from Torlak, Serbia. Agar, anhydrous glucose, and yeast extract were purchased from Thermo Fisher Scientific (United Kingdom). Gallium(III) chloride, gallium(III) sulfate, barium chloride dihydrate, sodium hydroxide, sodium chloride, hydrochloric acid, sulfuric acid, 1,3-propanediamine, (±)-1,3-pentanediamine, chloroacetic acid, phosphate-buffered saline (PBS), dimethyl sulfoxide (DMSO), BSA, ct-DNA, ethidium bromide (EthBr), 2′-(4-hydroxyphenyl)-5-[5-(4-methylpiperazine-1-yl)benzimidazo-2-yl]-benzimidazole (Hoechst 33258, Hoe), eosin Y (eos Y), ibuprofen (ibu), digitoxin (dig), ethanol, and deuterated water (D_2_O) were purchased from the Sigma-Aldrich Chemical Co (Steinheim, Germany).

Elemental analysis for carbon, hydrogen, and nitrogen was performed using a NC Technologies ESC 8020 Organic Elemental Analyzer. The pH measurements were done at room temperature using the pH meter S220 SevenCompact pH/Ion, Mettler Toledo, previously calibrated with the buffer solution of pH 4.01 and 7.00. The IR spectra were recorded on a PerkinElmer Spectrum Two FT-IR spectrometer over the wavenumber range of 4000–450 cm^−1^ using the KBr pellet technique. The NMR spectra in D_2_O were recorded on a Bruker Avance III 600 MHz spectrometer in a 5-mm NMR tube at room temperature. Chemical shifts, *δ*, are expressed in ppm, and scalar couplings, *J*, are given in Hz. ^1^H NMR spectroscopy was also used to investigate the solution stability of gallium(III) Complex **1** after its dissolution in D_2_O, as well as after 48 h at room temperature. The molar conductivity was measured on a conductometer Hanna Edge Conductivity Meter at room temperature. The concentration of the aqueous solution of Complex **1** used for conductivity measurements was 1 × 10^−3^ M. The interactions of Complex **1** with BSA/ct-DNA were investigated by fluorescence emission spectroscopy using Jasco FP-6600 spectrofluorometer.

### 2.2. Synthesis of 1,3-H_2_pdda^.^2HCl

1,3-Propanediamine-*N,N′*-diacetic acid (1,3-H_2_pdda) was obtained using a slightly modified procedure that was previously published [10]. 0.2 mol of chloroacetic acid (18.9 g) was dissolved in 25.0 mL of water and cooled in an ice bath. 0.2 mol of NaOH (8.0 g) dissolved in 16.0 mL of water was added to this solution, dropwise under stirring, keeping the temperature in the range of 10–15°C. After the addition of NaOH solution, 0.1 mol (7.4 g; 8.4 mL) of 1,3-propanediamine was added to the reaction mixture, and then, 0.2 mol (8.0 g) of NaOH dissolved in 16.0 mL of water was added in small portions. The stirring continued for the next 4 h, while the temperature was kept between 80°C and 90°C. The obtained reaction mixture was neutralized with 28.0 mL of 6.0 M hydrochloric acid. The volume of the resulting solution was reduced to 35.0 mL, and then, an additional volume of concentrated HCl (ca. 30 mL) was added with stirring. Deposited NaCl was removed by filtration. After that, 30.0 mL of concentrated HCl, 30.0 mL of ethanol, and 30.0 mL of ether were added to the filtrate. The resulting mixture was cooled in an ice bath for 4 h. After cooling, the precipitate was separated by filtration. The crude product was recrystallized from a water/ethanol mixture (v/v, 1 : 1). The yield of the reaction for the synthesis of ligand was 68% (17.9 g).

The purity of 1,3-H_2_pdda^.^2HCl before its use for the preparation of Complex **1** was checked by elemental analysis. Elemental analysis for 1,3-H_2_pdda^.^2HCl = C_7_H_16_Cl_2_N_2_O_4_ (MW = 263.12 g/mol): found: C, 31.86; H, 6.09; and N, 10.49%; calc.: C, 31.95; H, 6.13; and N, 10.65%.

### 2.3. Synthesis of Uns-*cis*-[Ga(1,3-pdda)(µ-OH)]_2_^.^2H_2_O (**1**)

1.0 mmol of GaCl_3_ (176.1 mg) was dissolved in 10.0 mL of water at 80°C. To this solution, 3.0 mmol of NaOH (120.0 mg) dissolved in 5.0 mL of water was added slowly under stirring. After the dissolution of the obtained white precipitate, 1.0 mmol of 1,3-H_2_pdda^.^2HCl (263.1 mg) was added to the reaction mixture. The stirring continued for the next 3 h at 80°C. After cooling at room temperature, 5.0 mL of ethanol was added to this solution and the obtained mixture was left in a refrigerator. Colorless crystals of uns-*cis*-[Ga(1,3-pdda)(*µ*-OH)]_2_·2H_2_O were formed after 3 weeks. These crystals were collected by filtration, washed with ethanol, and air-dried. The yield of Complex 1 was 39% (114.2 mg).

Elemental analysis for **1** = C_14_H_30_Ga_2_N_4_O_12_ (MW = 585.86 g/mol): found: C, 28.51; H, 5.03; and N, 9.64%; calc.: C, 28.70; H, 5.16; and N, 9.56%. IR (KBr, *ν*, cm^−1^): 3466br (*ν* (O–H)), 3261m (*ν* (N–H)), 2985w, 2948w, 2918w (*ν* (C–H)), 1656m, 1623vs (*ν*_*as*_ (COO^−^)), 1394m, 1384m, 1376m, 1366m (*ν*_*s*_ (COO^−^)). ^1^H NMR (600 MHz, D_2_O): *δ* = 3.69 (*s*, 2H, C4H), 3.35–2.88 (*m*, 2H, C1H/C3H), 2.14–1.75 (*m*, 1H, C2H) ppm. ^13^C NMR (150 MHz, D_2_O): *δ* = 170.31 (C5), 48.64 (C4), 44.23 (C1/C3), 22.46 (C2) ppm. Λ_*M*_ (H_2_O): 49.5 Ω^−1^cm^2^mol^−1^.

### 2.4. Synthesis of Na[Ga(1,3-pdta)]·3H_2_O **(2)** and Ba[Ga(1,3-pndta)]_2_·3H_2_O **(3)**

1,3-Propanediamine-*N,N,N′,N′-*tetraacetate (1,3-pdta^4−^) and (±)-1,3-pentanediamine-*N,N,N′,N′*-tetraacetate (1,3-pndta^4−^) were used as ligands for the synthesis of Na[Ga(1,3-pdta)]·3H_2_O (**2**) and Ba[Ga(1,3-pndta)]_2_·3H_2_O (**3**) in accordance with a previously published method [34]. The purity of these complexes was checked by elemental analysis and ^1^H NMR spectroscopy. The obtained data were in agreement with those for the previously characterized gallium(III) complexes [34].

Elemental analysis for **2** = C_11_H_20_GaN_2_NaO_11_ (MW = 449.00 g/mol): found: C, 29.36; H, 4.55; and N, 6.15%; calc.: C, 29.43; H, 4.49; and N, 6.24%. IR (KBr, *ν*, cm^−1^): 3433br (*ν* (O–H)), 2985w, 2972w, 2945w (*ν* (C–H)), 1678vs, 1646vs, 1626vs (*ν*_*as*_ (COO^−^)), 1370vs, 1335vs (*ν*_*s*_ (COO^−^)). ^1^H NMR (600 MHz, D_2_O): *δ* = 4.10–3.53 (4CH_2_, G/R rings), 3.41–2.86 (*m*, 2H, C1H/C3H), 2.08 (*m*, 1H, C2H) ppm. ^13^C NMR (150 MHz, D_2_O): *δ* = 175.01 (C5 (2R)), 174.18 (C5′ (2G)), 63.80 (C4′ (2G)), 57.90 (C4(2R)), 52.93 (C1/C3), 19.61 (C2) ppm. Λ_*M*_ (H_2_O): 86.8 Ω^−1^cm^2^mol^−1^.

Elemental analysis for **3** = C_26_H_42_BaGa_2_N_4_O_19_ (MW = 991.41 g/mol): found: C, 31.38; H, 4.35; and N, 5.72%; calc.: C, 31.50; H, 4.27; and N, 5.65%. IR (KBr, *ν*, cm^−1^): 3439br (*ν* (O–H)), 2989w, 2969w, 2945w (*ν* (C–H)), 1639vs (*ν*_*as*_ (COO^−^)), 1370s (*ν*_*s*_ (COO^−^)). ^1^H NMR (600 MHz, D_2_O): *δ* = 4.04–3.28 (*m*, 4CH_2_, G/R rings), 2.91 (*m*, 2H, C1H), 2.29 (*m*, 1H, C3H), 1.88–1.36 (*m*, 2H, C2H/C4H), 0.83 (*m*, 3H, C5H) ppm. ^13^C NMR (150 MHz, D_2_O): *δ* = 175.14, 175.06 (C7 (R1, R2)), 174.15 (C7′ (2G)), 64.16, 63.56 (C6′ (G1, G2)), 62.50, 57.19 (C6 (R1, R2)), 54.05 (C3), 52.13 (C1), 25.49 (C2), 23.72 (C4), 9.59 (C5) ppm. Λ_*M*_ (H_2_O): 262.3 Ω^−1^cm^2^mol^−1^.

### 2.5. Crystal Structure Determination

Single-crystal X-ray diffraction data for **1** were collected on an Agilent Technologies SuperNova Dual diffractometer with an Atlas detector at 150 K using monochromated Mo-Kα radiation (*λ* = 0.71073 Å). The obtained data were processed using CrysAlis Pro [36]. The structure was solved by the SHELXT program [37] and refined by a full-matrix least-squares procedure based on *F*^2^ with SHELXL [38] using the Olex2 program suite [39]. All non-hydrogen atoms were refined anisotropically. All the hydrogen atoms were readily located in different Fourier maps, and H atoms bonded to C atoms were subsequently treated as riding atoms in geometrically idealized positions with *U*_*iso*_(H) = 1.2*U*_*eq*_(C). H atoms bonded to N, and O atoms were treated by fixing N/O–H bond lengths with *U*_*iso*_(H) = 1.2*U*_*eq*_(N) and *U*_*iso*_(H) = 1.5*U*_*eq*_(O). The crystallographic data are listed in Table S1. Cif and CheckCif files for Complex **1** are provided under the Supporting section.

### 2.6. Antimicrobial Activity

#### 2.6.1. Determination of Minimum Inhibitory Concentrations (MICs)

MIC values of gallium(III) Complexes **1**–**3**, 1,3-H_2_pdda^.^2HCl, 1,3-H_4_pdta, 1,3-H_4_pndta, and corresponding gallium(III) salts were determined according to the standard microdilution assays in accordance to the Standards of European Committee on Antimicrobial Susceptibility Testing (v 7.3.1: Method for the determination of broth dilution MICs of antifungal agents for yeasts) for *Candida* spp., and according to the standard broth microdilution assays, recommended by the National Committee for Clinical Laboratory Standards (M07-A8) for bacteria.

The tested microorganisms included four bacterial strains (*Pseudomonas aeruginosa* PAO1, *Escherichia coli* NCTC 9001, *Staphylococcus aureus* ATCC 25923, and *Listeria monocytogenes* NCTC 11994) and one fungal strain (*Candida albicans* ATCC 10231) obtained from culture collections (ATCC—American Type Culture Collection, NCTC—National Collection of Type Cultures). Bacterial stock cultures were revived on Luria-Bertani agar plates (LB: 10 g/L NaCl, 5 g/L yeast extract, 10 g/L tryptone) and fungal cultures were revived on Sabouraud plates (SAB: 40 g/L glucose, 10 g/L peptone, 20 g/L agar) at 37°C overnight. The tested compounds were dissolved in water at a concentration of 50 mM. The highest used concentration was 500 μM. The inoculums were 1 × 10^5^ colony-forming units (cfu)/mL for fungal cultures and 5 × 10^5^ cfu/mL for bacteria. Inhibition of bacterial growth was determined by measuring absorbance at 625 nm (OD_625_) for bacteria and at 530 nm (OD_530_) for fungi, using a plate reader (Epoch Microplate Spectrophotometer, Bio Tek Instruments, Inc., USA). The negative control (media only) and positive control (microorganisms without inhibitors in liquid broth) on the same plate were used as references to determine the growth inhibition of bacteria.

#### 2.6.2. Anti-Quorum Sensing (QS) Activity

We have also investigated the anti-QS activity of Complexes **1**–**3** using different biosensor strains, *Chromobacterium violaceum* CV026, *Serratia marcescens* ATCC 27117, and *Pseudomonas aeruginosa* BK25H. The assessment of the violacein production in *C. violaceum* CV026 was done according to the previously described methodology [40]. This strain was cultivated in LB growth medium, supplemented with appropriate antibiotics overnight at 30°C and 180 revolutions per minute (rpm) on a rotary shaker. Into semisolid LB agar (0.3%, w/v, 5.0 mL), 50.0 μL of an overnight culture of *C. violaceum* CV026 supplemented with *N*-hexanoyl-(L)-homoserine lactone (Sigma, Germany) to a final concentration of 5.0 μM was seeded and finally poured over the surface of LB agar plates. *N*-hexanoyl-(L)-homoserine lactone was used because this biosensor strain is not capable to produce autoinducer for QS signaling pathway, and for this assay, it is added externally. After solidification, the sterile discs were placed into the surface of plates and complexes were added in appropriate concentrations (100 μg per disc). Petri dishes were incubated at 30°C in upright position overnight.

For the prodigiosin production, *Serratia marcescens* ATCC 27117 was used. The assay was assessed similarly, except that no external autoinducer was added, as this wild-type strain naturally produces it [41]. The inhibition of violacein and prodigiosin production was indicated by the presence of hazy hallows around discs containing complexes.

Furthermore, the assay used for the determination of pyocyanin production was performed using the previously published method [42] with some minor modifications. *Pseudomonas aeruginosa* BK25H strain was cultivated overnight and that culture was subcultured 1:1000 into 5 mL of fresh LB medium. The tested compounds were assayed at a concentration of 100 μg/mL (subinhibitory concentration). The incubation period was 20 h at 37°C on a rotary shaker at 180 rpm. OD_600_ was measured for whole cultures, and cells were separated from culture fluids by centrifugation at 14,000 rpm for 10 min. OD_695_ of supernatants was measured using a UV-1900i UV–Vis spectrophotometer (Shimadzu, Japan). The values of OD_695_ were normalized per cell density.

#### 2.6.3. Inhibition of *P. aeruginosa* PAO1 Biofilm Formation

Biofilm quantification assays were performed in microtiter plates using a crystal violet staining method to evaluate adherent cells [43]. Overnight cultures of *P. aeruginosa* strains were inoculated in TSB medium (Tryptic soy broth, HiMedia Laboratories) at 37°C, with and without the tested compounds in a 96-well microtiter plate. After 24 h, free (detached) cells were removed, and wells were washed with PBS. Biofilms were fixed with 100 mL of 99% (v/v) methanol, followed by staining with 0.4% (v/v) crystal violet. After washing, crystal violet was solubilized with 150 μL of glacial acetic acid (33%, v/v) and absorbance was measured at 540 nm.

#### 2.6.4. Interference of the Gallium(III) Complexes With QS Pathways

The pathogenicity of *P. aeruginosa* depends on the coordinated expression of virulence factors, largely regulated by QS. *P. aeruginosa* has a complex QS network with three systems that rely on the production of the specific signaling molecules: 3OC12-HSL3OC12-HSL (*N*-3-oxo-dodecanoyl-(L)-homoserine lactone), C4-HSL (*N*-butyryl-(L)-homoserine lactone), and AHQs (2-alkyl-4-quinolones) [44, 45]. In this study, three biosensor strains were developed to identify which QS pathways were affected by the gallium(III) Complexes **1**–**3**. The PA14-R3ΔlasIPrsaI: lux strain was used to measure the levels of 3OC12-HSL, while PAO1 ΔrhlIpKD-rhlA was employed to quantify C4-HSL in *P. aeruginosa* culture supernatants. The final biosensor PAO1 ΔpqsA mini-CTX luxPpqsA was utilized to evaluate the impact of Complexes **1**–**3** on the production of AHQs in *P. aeruginosa* PAO1. Overnight cultures of these biosensor strains were diluted to an optical density of 0.045, measured at 600 nm (OD_600_), and incubated with complexes (0.5 × MIC) in the presence of 5 μM of the corresponding specific autoinducers for 4 h at 37°C on a rotary shaker at 70 rpm. OD_600_ and bioluminescence were simultaneously measured using a Tecan Infinite 200 Pro multiplate reader (Tecan Group Ltd., Männedorf, Switzerland). Luminescence values were normalized per cell density.

### 2.7. Cytotoxicity Assay

Cytotoxicity of Complexes **1**–**3**, 1,3-H_2_pdda^.^2HCl, 1,3-H_4_pdta, 1,3-H_4_pndta, and the corresponding gallium(III) salts was determined as antiproliferative effect on human fibroblasts MRC-5 (obtained from ATCC), as described previously [46, 47]. Cytotoxicity was assessed by 3-(4,5-dimethylthiazol-2-yl)-2,5-diphenyltetrazolium bromide (MTT) colorimetric assay. The assay was carried out after 48 h of cell incubation in media (RPMI (Roswell Park Memorial Institute) 1640 medium, Gibco by Thermo Fisher Scientific CE, supplemented with 10% fetal bovine serum (FBS), 100 U/mL penicillin, and 100 μg/mL streptomycin) containing the studied compounds at concentrations ranging from 200 to 25 μM. Cell proliferation was determined by absorbance at 540 nm on a multiplate reader (Epoch 2000, Bio Tek, Winooski, USA). The MTT assay was performed in quadruplicate, and results were expressed as percentages of the control (untreated cells) that was arbitrarily set to 100%. The cell viability rate (%) was calculated as follows: (A of the treated group/A of the control group) × 100. The results are presented as a percentage of the control (cells treated with) that was arbitrarily set to 100%.

### 2.8. BSA Binding Study

The stock solution of Complex **1** was prepared in water (1.0 × 10^−2^ M). The BSA binding study was performed by tryptophan fluorescence quenching experiments in PBS (pH = 7.4) at room temperature in the range of 295–500 nm, with an excitation wavelength of 290 nm. The concentration of BSA was constant (5 μМ), while the concentration of Complex **1** (0–88 μM) gradually increased.

The following equation was used for the calculation of the Stern–Volmer constants (*K*_*sv*_) [48]:

(1) $$\begin{array}{c} \frac{F_{0}}{F}=1+K_{q}\tau_{0}\left[\text{complex}\right]=1+K_{sv}\left[\text{complex}\right]. \end{array}$$

*F*_0_ and *F* are the fluorescence intensities in the absence and presence of the complex, respectively. *K*_*q*_ represents the bimolecular quenching constant, and *τ*_0_ is the lifetime (10^−8^ s) of the fluorophore in the absence of the complex. The binding constants (*K*_*A*_) and the number of binding sites (*n*) can be calculated using the following equation [49]:

(2) $$\begin{array}{c} \frac{\log\left(F_{0}-F\right)}{F}=\log\;K_{A}+n\;\log\left[\text{complex}\right]. \end{array}$$

We also studied the competitive BSA interactions of Complex **1** with the site markers, eosin Y (eos Y, a marker for Site I of the subdomain IIA), ibuprofen (ibu, a marker for Site II of the subdomain IIIA), and digitoxin (dig, a marker for Site III of the subdomain IB). The fluorescence emission range and the excitation wavelength were as defined above. The BSA and markers were mixed in equimolar amounts (8 μM), and the increased concentrations of the Complex **1** were added (0–149 μM).

### 2.9. ct-DNA Binding Study

A stock solution of ct-DNA was prepared by dissolving the solid substance in PBS. The concentration of the obtained solution (1.30 × 10^−2^ M) was determined from UV absorbance at 258 nm using the molar extinction coefficient *ε* = 6.6 × 10^3^ M^−1^cm^−1^ [50]. The stock solutions of EthBr (1.01 × 10^−2^ M) and Hoechst 33258 (1.0 × 10^−2^ M) were prepared in DMSO, while the solution of the investigated complex (1.0 × 10^−2^ M) was prepared in water.

The ct-DNA binding experiments were performed in PBS by keeping a [ct-DNA] : [EthBr/Hoe] ratio of 10 : 1, while gradually increasing the concentration of Complex **1** (0–69 μM). The competitive binding study between EthBr and the complex toward ct-DNA was followed in the range of 525–750 nm, with the excitation wavelength of 520 nm, while the competitive interaction between Hoe and the complex toward ct-DNA in the range of 351–750 nm, with the excitation wavelength of 346 nm. The *K*_*sv*_, *K*_*q*_, and *K*_*A*_ constants, and the *n* number were calculated as described above for BSA binding experiment [48, 49].

### 2.10. HSA

The objective of this study is to evaluate the intermolecular interactions that impact the stability of the crystal structure by the approach of Hirschfeld surface analysis (HSA). The present methodology enables the identification and evaluation of the relative impact of several categories of interactions occurring within the crystal. The selection of HSA was based on its capacity to offer both visual and quantitative observations of the interactions occurring within the crystal. This facilitates the comprehension of how individual atoms and molecules contribute to the stabilization of the complex structure. The research was conducted using Crystal Explorer 21.5 [51], a highly dependable software for Hirshfeld analysis known for its exceptionally accurate generation of three-dimensional surfaces and two-dimensional fingerprint plots. The input data for the analysis of Complex **1** were obtained from a crystallographic information file (CIF). The normalized contact distance (*d*_*norm*_) is the crucial metric in HSA that allows for the detection of certain domains in the crystal structure where the most important intermolecular interactions are established. This parameter is determined by calculated values of the real distances between atoms and their van der Waals radii. The numerical expression for *d*_*norm*_ is as follows:

(3) $$\begin{array}{c} d_{\text{norm}}=\frac{\left(d_{i}+r_{i}^{vdW}\right)}{r_{i}^{vdW}}+\frac{\left(d_{e}-r_{e}^{vdW}\right)}{r_{e}^{vdW}}, \end{array}$$

where *r*_*i*_^*vdW*^ and *r*_*e*_^*vdW*^ are the van der Waals radii of the atoms inside and outside the surface, respectively, and *d*_*i*_ and *d*_*e*_ are the distances between the surface and the nearest atom inside and outside the surface [51, 52]. Three-dimensional *d*_*norm*_ surfaces were created using a color plan that graphically depicts the contact interactions: red denotes strong interactions (lower *d*_*norm*_ values), while blue indicates weaker interactions. The presence of these surfaces facilitates the visual identification of important interactions within the crystal and enhances the intuitive understanding of the intermolecular forces involved. Furthermore, alongside the three-dimensional surfaces, two-dimensional “fingerprint plots” were generated. The presented graphs depict the correlation between the internal (*d*_*i*_) and external (*d*_*e*_) distances, thereby facilitating a quantitative examination of diverse forms of contacts. The “fingerprint” analysis enables the quantifying of the individual impact of each contact on the stability of the crystal, therefore offering a comprehensive understanding of the intermolecular forces that maintain the crystal structure.

### 2.11. Computational Methodology

This work utilized the *Gaussian16* software package [53] in conjunction with the *GaussView 6.0.16* program [54] to conduct quantum-mechanical calculations based on density functional theory (DFT) and provide graphical representations of molecular structures. For this analysis, the B3LYP-D3-BJ functional and the 6-311 + G (d, p) basis set were employed for all atoms, except the Ga atom, which utilized the LANL2TZ(f) basis set. It has been demonstrated that the theoretical method used provides an accurate description of the molecular structures [55, 56]. Gibbs free energy reaction (Δ_r_*G*^0^) for the hydrolysis process was calculated by optimizing the geometries of both reactants (*G*°_reactants_) and products (*G*°_products_) at this level of theory [57]. This study utilized the conductor-like polarizable continuum model (CPCM) to assess the impact of water as a solvent on the geometric characteristics of the studied complex [58]. Vibrational spectra of the complexes in the gas phase were analyzed using potential energy distribution (PED) analysis with FCART version 7.0 software [59]. The chemical shifts of protons and carbons in ^1^H and ^13^C NMR spectra were determined using the gauge-independent atomic orbital (GIAO) technique, by utilizing water as the solvent [60].

### 2.12. Molecular Docking Study

To thoroughly evaluate the binding affinity of the studied compounds with DNA and BSA macromolecules, a molecular docking study was employed.

The interactions of the studied compounds with BSA and DNA macromolecules were determined by molecular docking using AutoDock 4.2 software in conjunction with the Lamarckian genetic algorithm (LGA) [61]. The docking simulations utilized parameters including a maximum of 250,000 energy evaluations, 27,000 generations, and mutation and crossover rates set at 0.02 and 0.8, respectively. The docking procedure involved several steps: preparation of the ligand, configuration of the BSA and DNA macromolecules, and formation of the grid. Ligand structures were optimized using the specified theoretical level of theory. The three-dimensional X-ray crystallographic structure of BSA was retrieved from the RCSB Protein Data Bank (PDB ID: **4F5S**) [62]. Although chain B, residual atoms, heteroatoms, and water molecules were eliminated during the creation of BSA in BIOVIA Discovery Studio 4.0, only chain A was retained [63]. The search space for BSA was defined with a grid box of 60 × 60 × 60 Å and a grid spacing of 0.375 Å, covering the following XYZ coordinates: Site I (IIA): −4.80 × 30.50 × 101.01; Site II (IIIA): 10.91 × 16.30 × 119.72; Site III (IB): 19.86 × 33.53 × 97.92. Canonical B-DNA (6-bp hexanucleotide d(CGATCG)_2_ with the intercalative binding site [6-bp-DNA, PDB code: **1Z3F**] [64] and DNA dodecamer d(CGCGAATTCGCG)_2_ [10-bp-DNA, PDB code: **1BNA**]) [65] were also sourced from the RCSB Protein Data Bank. For molecular docking study with DNA molecules, the grid box dimensions were established as 50 × 50 × 50 Å (with a grid spacing of 0.375 Å) for the 6-bp-DNA structure, which is centered at 0.871 × 17.488 × 46.277 Å. For the 10-bp-DNA structure, the grid spacing was set to 60 × 74 × 120 Å with a grid spacing of 0.375 (centered at 15.81 × 21.31 × 9.88 Å). Other docking parameters were determined according to established procedures outlined in prior research [66–68].

## 3. Results and Discussion

### 3.1. Synthesis and Structural Characterization of Complex **1**

**Scheme 1:**
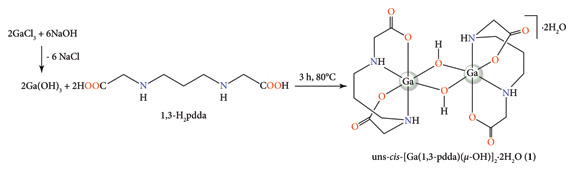
Schematic presentation of the synthesis of uns-*cis*-(Ga[1,3-pdda] [*µ*-OH])_2_^.^2H_2_O (**1**).

Gallium(III) hydroxide, resulted from the reaction of gallium(III) chloride with sodium hydroxide solution, and 1,3-propanediamine-*N,N′*-diacetic acid (1,3-H_2_pdda) were used for the synthesis of dinuclear gallium(III) complex, uns-*cis*-[Ga(1,3-pdda)(*µ*-OH)]_2_^.^2H_2_O (**1**; Scheme 1). This complex was crystalized from water/ethanol solution (v/v, 1 : 1) after cooling in a refrigerator for 3 weeks. The purity of the complex was checked by elemental analysis. Coordination of the 1,3-pdda^2−^ ligand to Ga(III) ion was confirmed by IR, ^1^H, and ^13^C NMR spectroscopy, while the single-crystal X-ray diffraction analysis was used for the determination of the crystal structure of the complex obtained.

#### 3.1.1. Single-Crystal X-Ray Diffraction Structure Determination and Analysis

**Figure 2:**
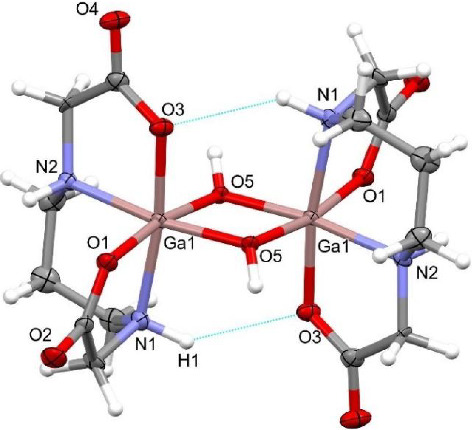
Dinuclear complex moiety in **1**. Thermal ellipsoids are drawn at the 50% probability level.

Complex **1** crystallizes in monoclinic *P*2_1_/*c* space group and contains half of dinuclear molecule and one water molecule in the asymmetric unit as a consequence of the presence of the inversion center. Gallium(III) ion is octahedrally coordinated by 1,3-pdda^2–^ ligand through two carboxylate oxygen atoms and two nitrogen atoms of secondary amine, and two oxygen atoms from bridging hydroxyl groups forming complex of uns-*cis* configuration (Figure 2). The Ga–O bond lengths (1.984(2) and 1.970(3) Å) and Ga–N bond lengths (2.096(3) and 2.070(3) Å) are comparable with those observed in the s-*cis*-[Ga(edda)(Leu)]_2_[{Ga(edda)(*μ*-OH)}_2_]·6H_2_O complex (Leu is amino acid (L)-leucine) [35]. Dinuclear unit is formed through two hydroxide bridging ligands with Ga–O bond lengths of 1.949(2) and 1.939(2) Å and is supported by intramolecular N1–H1⋯O3 interaction (Figure 2 and Tables S2 and S3). In Complex **1**, the central Ga_2_O_2_ ring is planar with bridging angle Ga1–O5–Ga1^i^ of 99.6(1)° and Ga1⋯Ga1^i^ separation of 2.9697(5) Å (where *i* is 1 − *x*, −y, 2 − z). The out-of-plane deviation of the bridging hydroxyl groups is 49.7° measured as the angle between Ga_2_O_2_ plane and the O–H bond. The conformations of the chelate rings are *chair* for the 1,3-propanediamine ring, *envelope* for the G (in-plane) glycinate ring and *twist* for the R (out-of-plane) glycinate ring (Table S4) [69]. The *chair* conformation of the 1,3-propanediamine ring has also been observed in the [Co(1,3-pdda)(*µ*-H_2_O)]_2_·4H_2_O [70] (Table S4) and [Cr(1,3-pdda)(mal)]^−^ [16] (mal is malonato) complexes of uns*-cis* configuration. The results of analysis of octahedral distortion in dinuclear six-coordinate 1,3-pdda^2−^ complexes of gallium(III) and cobalt(II) [70] of uns*-cis* configuration are given in Table S5. Complex **1** shows similar distortion of the octahedral bond angles as the [Ga(1,3-pdta)]^−^ complex anion [34], as indicated by comparison of the ∑Δ(*O*_*h*_) values (52° vs. 53° and 51°, respectively). The mean angular deviation for each complex is nearly four degrees per angle. The deviation of the endocyclic bond angles in the glycinate ring of the R type is comparable with that observed for the same kind of rings in [Ga(1,3-pdta)]^−^ [34], while the deviation of the endocyclic bond angles in the glycinate ring of the G type is significantly smaller compared to that observed for the same kind of rings in [Ga(1,3-pdta)]^−^ [34] (−2° vs. −9°). However, structurally similar uns*-cis*-[Co(1,3-pdda)(*µ*-H_2_O)]_2_·4H_2_O complex [70] shows significantly lower distortion of the octahedral bond angles than the [Co(1,3-pdta)]^2−^ [71], as indicated by comparison of the ∑Δ(*O*_*h*_) values (35° vs. 71°). No deviation of the R and G glycinate rings from the ideal (538.5°) bond angle sum was observed in the uns*-cis*-[Co(1,3-pdda)(*µ*-H_2_O)]_2_·4H_2_O (Table S5).

**Figure 3:**
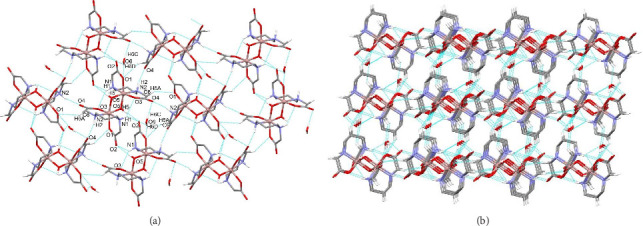
(a) Hydrogen-bonded layer along *bc-*plane in **1**. (b) 3D supramolecular formation. Hydrogen atoms not involved in the motif shown have been omitted for clarity.

A series of hydrogen bonding interactions enable the formation of a supramolecular framework. A hydrogen-bonded layer along *bc*-plane is formed through N1–H1⋯O2 and O5–H5⋯O2 interaction of 1,3-pdda^2−^ and hydroxide ligands with carbonyl O2 atom of the adjacent molecule as well as through N2–H2⋯O4 interactions of 1,3-pdda^2−^ ligand with carbonyl O4 atom of the adjacent molecule. This layer is supported by C6–H6A⋯O1 interactions of 1,3-pdda^2−^ ligand and carboxylic O1 atom of the adjacent molecule as well as by interactions of hydrate water molecule connecting two adjacent complex molecules through O6–H6C⋯O4 and bifurcated O6–H6D⋯O1/O2 hydrogen bonding (Figure 3 and Table S3). Layers are connected into 3D supramolecular structure through C2–H2B⋯O6 interaction between 1,3-pdda^2−^ ligand and water molecule as well as through C4–H4A⋯O3 interaction between 1,3-pdda^2−^ ligand and carboxylic O3 atom of adjacent molecule (Figure 3 and Table S3).

#### 3.1.2. HSA for Complex **1**

**Figure 4:**
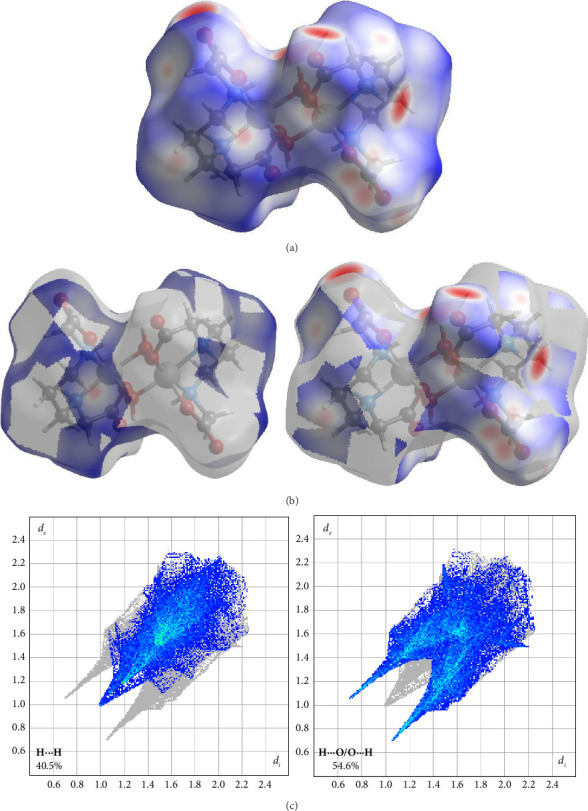
(a) Three-dimensional Hirshfeld surfaces mapped over *d*_norm_ for all interactions, (b) highlighting dominant H⋯H (left) and H⋯O/H⋯O (right) interactions, and (c) two-dimensional “fingerprint plots” showing the percentage contributions of the key interactions in the crystal structure of complex **1**.

The intermolecular interactions crucial to the stabilization of the structure of Complex **1** were identified using HSA (Figure 4). In the *d*_*norm*_ surfaces, the predominant contacts were identified, with H⋯O/O⋯H interactions making up 54.6% of the total interactions, and H⋯H contacts accounting for 40.5%. The results indicate that the crystal structure is crucially stabilized by hydrogen bonds between hydrogen and oxygen atoms, with substantial contribution from van der Waals interactions between hydrogen atoms.

The two-dimensional “fingerprint plots” further confirmed these findings by quantifying the different types of interactions (Figure 4(c)). The peaks observed in the fingerprints for H⋯H and H⋯O/H⋯O contacts provide important insights into the characteristics of these interactions. The H⋯H contacts displayed a distinct peak between the 1.0 to 1.2 Å region, suggesting extremely brief and direct interactions between hydrogen atoms. The presence of this clear and symmetric peak is characteristic of van der Waals interactions, which are quite feeble but uniformly dispersed throughout the crystal structure, thereby contributing to its stability. H⋯O/H⋯O interactions, representing 54.6% of all contacts, produced two symmetric peaks between the 0.7 and 1.4 Å region. These peaks exhibit a greater degree of sharpness in comparison with the H⋯H connections, indicating the existence of more robust but also more fluctuating interactions. The observed peaks correspond to hydrogen bonds, which are renowned for their robustness and significantly contribute to the crystal stability.

#### 3.1.3. Spectroscopic Characterization of Complex **1**

**Figure 5:**
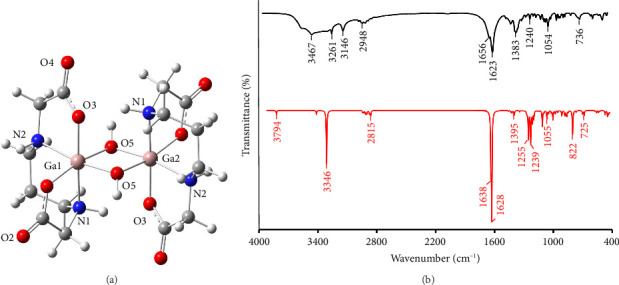
Optimized geometries of the complex **1** in the gas phase (a), along with the experimental (black line) and simulated (red line) IR spectra (b). Calculations were performed using the B3LYP-D3-BJ functional and the 6-311 + G (d, p) basis set for all atoms, except for Ga, where the LANL2TZ(f) basis set was applied. Legend: Ga—pink, C—grey, H—white, and O—red, N—blue.

The experimental and theoretical IR spectroscopic data for Complex **1**, recorded in the range of 4000–450 cm^−1^, were analyzed to gain deeper insight into its vibrational characteristics. To complement the experimental findings, an IR spectrum simulation was performed using the DFT method. The geometry of Complex **1** was first optimized in the gas phase, employing the B3LYP-D3-BJ functional and the 6-311 + G (d, p) basis set for all atoms, except the Ga atom, which utilized the LANL2TZ(*f*) basis set. By comparing the optimized geometric parameters, such as bond lengths and angles, with those obtained by X-ray analysis of Complex **1**, it was confirmed that the applied level of theory effectively describes the molecular structure of this complex (Figure 5). The high correlation factor (R) between the theoretical and experimental bond lengths (*R* = 0.940) and angles (*R* = 0.989) underscores the reliability of the chosen model (Table S2). The structure of Complex **1** is stabilized by intramolecular hydrogen bonding including O3⋯N1 (1.967 Å).

Figure 5 illustrates both experimental and computational IR spectra, measured across the wavenumber range of 4000 to 450 cm^−1^. The theoretical spectrum was derived from optimized geometry shown in Figure 5, where initial geometry was based on the crystal structure of Complex **1**. To address the typical overestimation of theoretical vibrational frequencies, a scaling factor was employed. This factor was determined as the square of the difference between the experimental and theoretical values, resulting in a value of 0.94701. It is noteworthy that the number of computed wavenumbers is significantly larger in the theoretical analysis.

When analyzing IR spectra, it is important to provide a detailed overview, focusing on the characteristic vibrational modes. In this study, three distinct regions were observed in both the experimental and theoretical spectra. The first region, ranging from 4000 to 2800 cm^−1^, is dominated by stretching vibrations. The broad band in the range 3467–2948 cm^−1^ in the experimental spectrum can be attributed to the superposition of O–H and N–H stretching vibrations, along with the contribution of C–H stretching vibrations. In the theoretical spectrum, O–H stretching vibrations are positioned at a higher frequency, around 3794 cm^−1^, while N–H stretching vibrations appear at 3346 cm^−1^, and different stretching C–H vibrations at 2815 cm^−1^. The difference between the experimental and theoretical wavenumbers is attributed to the fact that the simulated spectra consider isolated geometries in the gaseous phase, which do not account for intermolecular interactions present in the experimental conditions. Despite these differences, the alignment of key peaks, especially those involving O–H and N–H vibrations, supports the accuracy of the computational approach.

The second region encompasses bands in the range of 1600–1000 cm^−1^, primarily arising from C=O stretching vibrations, with additional contributions from C–C–C, H–C–C, and H–C–H bending vibrations. The position of asymmetric stretching frequencies of carboxylate groups has been previously successfully employed as criteria for distinguishing between unionized and uncoordinated –COOH groups (1750–1700 cm^−1^) and ionized and coordinated COO^−^ groups (< 1700 cm^−1^) [16, 18, 33, 72–75]. Additionally, the analysis of IR spectra in the carboxylate region could be successfully applied for the determination of the type of geometrical isomers of octahedral edda^2−^ and 1,3-pdda^2−^ transition metal complexes [16, 18, 72].

In the IR spectrum of Complex **1,** two bands at 1656 and 1623 cm^−1^ were observed in the asymmetric carboxylate stretching frequency region, indicating that all carboxylate groups of 1,3-pdda^2−^ ligand are coordinated to the gallium(III) ion (Table S6). On the other hand, these bands are positioned in the theoretical spectrum at 1638 and 1628 cm^−1^. IR spectrum of Complex **1** is compared with those previously reported for Complexes **2** and **3** containing hexadentately coordinated 1,3-pdta^4−^ and 1,3-pndta^4−^ ligands, respectively [34] (Table S6). The latter two complexes in the region for asymmetric stretching vibrations of carboxylate groups showed one intense band at 1646 cm^−1^ for **2** (with a tendency for the splitting at 1678 and 1626 cm^−1^) and at 1639 cm^−1^ for **3**. It is worth mentioning that the hexadentate coordination of 1,3-pdta^4−^ and 1,3-pndta^4−^ ligands and the octahedral geometry of the corresponding gallium(III) complexes have been confirmed by X-ray diffraction analysis [34]. On the other hand, in the IR spectrum of 1,3-H_2_pdda^.^2HCl, which is used for the synthesis of Complex **1**, only one intense band at 1746 cm^−1^ is observed, indicating the presence of protonated –COOH groups (Table S6). The presence of two bands due to the asymmetric stretching vibrations of carboxylate groups in the spectrum of **1** indicates that this complex represents uns-*cis* geometrical isomer as a consequence of nonequivalence of the axial and equatorial glycinate chelate rings [16, 18, 72]. This is in accordance with IR spectroscopic behavior of uns-*cis*-[Cr(1,3-pdda)(ox)]^.^H_2_O (1650 and 1684 cm^−1^) and uns-*cis*-[Cr(1,3-pdda)(mal)]·8H_2_O (1615 and 1660 cm^−1^) complexes (ox and mal are bidentate oxalate and malonate anions) [16]. Contrary to this, in the spectra of s-*cis*-[Cr(edda)(ox)]^.^H_2_O and s-*cis*-[Cr(edda)(mal)]^.^3H_2_O, only one band in the asymmetric carboxylate stretching frequency region was observed at 1640 and 1635 cm^−1^, respectively, due to their *C*_2_ symmetry [16].

The third region comprises bands with wavenumbers below 1000 cm^−1^, which are primarily attributed to torsional vibrations. These include H–C–C–H, C–C–C–H, H–C–N–C, and C–C–O–Ga torsional modes. These low-frequency vibrations arise from twisting motions within the molecular framework and are often sensitive to the overall molecular conformation. A comparison between the experimental and theoretical spectra shows a good alignment of these torsional vibrations, indicating that the theoretical model effectively captures the structural characteristics of the investigated compounds.

NMR (^1^H and ^13^C) spectra of Complex **1** were recorded in D_2_O at room temperature (Figures S2 and S3). Theoretical NMR spectra (^1^H and ^13^C) were generated in water using the GIAO protocol at the B3LYP-D3-BJ/6-311++G (d, p) level of theory. The chemical shifts of the simulated figures are shown in Table S7. The strong correlation (> 0.993) seen between the experimental and theoretical chemical shift values suggests that the theoretical model used effectively characterizes the structure of the molecule in an aqueous solution. This agreement validates the stability of the structure of Complex **1** in the aqueous solution and the suitability of the theoretical methodology employed.

The experimental ^1^H NMR spectrum of Complex **1** exhibits three unique chemical shift areas for protons (Figure S2), which correspond to their relative chemical surroundings. Two multiplets in the regions 2.14–1.75 ppm (theoretical spectrum 1.89 ppm) and 3.35–2.88 ppm (theoretical spectrum 3.22 ppm) are assigned to the C2H and C1H/C3H protons of the 1,3-propanediamine ring, respectively. The lower chemical shift of the C2H protons results from greater shielding due to their proximity to the central part of the ring, where the influence of neighboring electronegative groups is weaker. In contrast, the C1H and C3H protons exhibit higher chemical shifts because they are closer to the terminal amine groups, leading to increased deshielding effects from the greater delocalized electron influence. A singlet at 3.69 ppm, corresponding to the theoretical spectrum of 3.41 ppm, is attributed to the methylene protons of the glycinate rings. The larger chemical shift can be attributed to the nearby carboxylate group, which enhances electron density and causes further deshielding of the protons. Deviations between experimental and theoretical chemical shift values are minimal (less than 0.3 ppm), indicating the high accuracy of the theoretical simulations. Since the spectrum was recorded in D_2_O, NH, and OH protons are not observable due to the deuterium exchange.

The ^13^C NMR spectrum of Complex **1** shows similar agreement between experimental and theoretical values (Figure S3 and Table S7). The chemical shift at 22.46 ppm (theoretical spectrum 21.15 ppm) is assigned to the central C2 carbon of the 1,3-propanediamine ring, while the shift at 44.23 ppm (theoretical spectrum 49.29 ppm) is assigned to the terminal C1 and C3 carbons of the same ring. The higher chemical shifts of these carbons indicate greater deshielding, due to the influence of adjacent amine groups. The chemical shift at 48.64 ppm (theoretical spectrum 48.20 ppm) is assigned to the methylene C4 carbon of the glycinate fragment, and the shift at 170.31 ppm (theoretical spectrum 174.31 ppm) is assigned to the C5 carbon of the carboxyl group of the glycinate rings. This high chemical shift is typical for carbonyl carbons, where delocalization of electron density through the double bond results in significant deshielding. The minimal deviation between experimental and theoretical values (less than 5 ppm) demonstrates that the theoretical model successfully predicts the electronic effects in the system. Both ^1^H and ^13^C NMR spectroscopic data for Complex **1** follow those for [Pt(1,3-pdda)]·3H_2_O complex, which was previously characterized by spectroscopic and crystallographic methods [76].

### 3.2. Stability in Solution

The behavior of Complex **1** in water, a solvent that was used for the preparation of stock solution for biological evaluation and its interaction with biomolecules, was investigated using ^1^H NMR spectroscopy and molar conductivity measurements. Complex **1** was dissolved in D_2_O and ^1^H NMR spectra were recorded immediately after dissolution, as well as after 48 h at room temperature (Figure S4). The position of the resonances in the ^1^H NMR spectra of the Complex **1** remained unchanged, indicating that 1,3-pdda^2−^ remains coordinated with the gallium(III) ion during the investigated time. The same is true for the Complexes **2** and **3**, in which no release of 1,3-pdta^4−^ and 1,3-pndta^4−^ ligands, respectively, from the gallium(III) ion was observed during 48 h [34].

The value of molar conductance for Complex **1** is given in the Materials and Methods section, and it is in accordance with its nonelectrolytic nature in water [77, 78]. Moreover, no significant change of the values of molar conductance of the complex was observed during 48 h.

**Figure 6:**
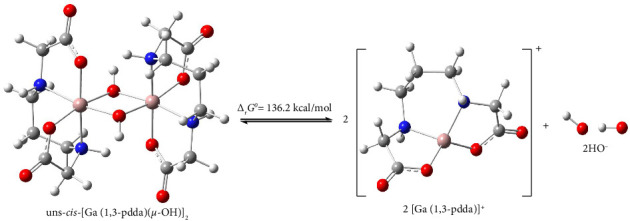
Optimized geometries of reaction participants in water optimized by B3LYP-D3-BJ functional and the 6-311 + G (d, p) basis set were employed for all atoms, except the Ga atom, which utilized the LANL2TZ(f) basis set and the hydrolysis process of complex **1** in water with Δ_r_*G*^0^ value. Legend: Ga—pink, C—grey, H—white, O—red, and N—blue.

The thermodynamic stability of the Complex **1** was evaluated using DFT by calculating the Gibbs free energy change (Δ_r_*G*^0^) for the reaction presented in Figure 6, under aqueous conditions modeled through the implicit solvent approach.

This approach to calculating Δ_r_*G*^0^ is recognized in the literature as a reliable method for assessing the stability of metal complexes, particularly in the context of reactivity in aqueous media, as it allows for a quantitative comparison of different complex systems at the thermodynamic level [79]. The calculated Δ_r_*G*^0^ values for the hydrolysis of Complex **1** show a distinctly endergonic character (136.2 kcal/mol), which suggests the high thermodynamic stability of this complex in an aqueous environment. The endergonic hydrolysis profile suggests that the Complex **1** is more energetically favored in the presence of water since a substantial energy input is necessary to break the bonds between the ligands and the central gallium(III) ion. This stability can be attributed to strong coordination interactions between gallium(III) and the ligands, as well as possible solvation effects, which further stabilize the complex. These findings are consistent with previous studies showing that gallium(III) ion forms highly stable complexes in aqueous media, particularly in the presence of ligands with a high affinity for metal ions, such as polydentate ligands [27].

### 3.3. Antimicrobial and Cytotoxic Studies

The antibacterial and antifungal activity was assessed for the gallium(III) Complexes **1**–**3**, salts used for their synthesis (GaCl_3_ and Ga_2_(SO_4_)_3_) and the corresponding aminocarboxylic acids (1,3-H_2_pdda^.^2HCl, 1,3-H_4_pdta, and 1,3-H_4_pndta) against selected bacterial strains (*E. coli*, *S. aureus*, *P. aeruginosa*, and *L. monocytogenes*) and one fungal strain (*C. albicans*). The antimicrobial activity of these compounds was characterized by MIC values, whereas the obtained results showed that Complexes **1**–**3** were inactive against all microorganisms at the highest concentration tested (MIC > 500 μM), except for *P. aeruginosa* strains. These complexes, along with the salts used for their synthesis (GaCl_3_ and Ga_2_(SO_4_)_3_), exhibited antibacterial activity against the *P. aeruginosa* PAO1 strain, with MIC values ranging from 31.25 to 125 μM for the complexes and 15.625–31.25 μM for the salts (Table 1). Furthermore, no activity was observed for aminocarboxylic acids against the tested pathogens. The present results for gallium(III) Complexes **1**–**3** are in contrast with those for previously reported gallium(III) complexes, including potassium *tris*(oxalato)gallate(III) trihydrate, *tris*(quinolono)gallium(III), and *tris*(3-hydroxy-2-methyl-4-pyronato)gallium(III) (gallium maltolate), as well as various gallium(III) complexes with pyridine-derived thiosemicarbazones and aroylhydrazones, which exhibited the antimicrobial potential against a range of Gram-negative and Gram-positive bacterial species. However, the results regarding the activity of **1**–**3** against *Pseudomonas* strains are consistent with the known activities of the mentioned complexes [80–86].

The antimicrobial activity of **1**–**3** was also compared with that of the previously synthesized chromium(III) and cobalt(III) complexes with 2,2-dimethyl-1,3-propanediamine-*N,N,N′,N′*-tetraacetato ligand (2,2-diMe-1,3-pdta^4−^), Na[Cr(2,2-diMe-1,3-pdta)]·3.75H_2_O, and Na[Co(2,2-diMe-1,3-pdta)]·3.88H_2_O [87]. As gallium(III) Complexes **1**–**3**, the latter two complexes did not inhibit the growth of *S. aureus* or *C. albicans*, even when a concentration of 500 μM was used. On the other hand, the complexation of metal(II) ions (Mn(II), Cd(II), Co(II), and Mg(II)) with 1,3-pdta^4−^ and 2,2-diMe-1,3-pdta^4−^ ligands results in selective anti-*Candida* activity of the obtained complexes [71, 88]. From this, it can be concluded that coordination of the aminocarboxylate ligands to metal(III) ion leads to increased difficulty in penetrating bacterial or fungal cell walls, thereby resulting in a decrease in antimicrobial activity.

Moreover, we examined the potential of Complexes **1**–**3**, salts used for their synthesis and the corresponding aminocarboxylic acids to modulate bacterial intercellular communication systems (QS), which regulates pathogenesis by controlling the expression of virulence factors [89]. Anti-QS activity of the mentioned compounds was tested using biosensor strains *C. violaceum* CV026 and *Serratia marcescens* ATCC 27117 at a concentration of 100 μg/disc. The presence of blurry white hallows around discs containing tested samples is an indication of the violacein and prodigiosin production controlled by QS. As can be seen from Figure S5, no activity of Complexes **1**–**3** on violacein production was observed, while only GaCl_3_ inhibited the violacein production, being in accordance with its anti-QS activity. The obtained results agree with those for chromium(III) and cobalt(III) complexes with 2,2-diMe-1,3-pdta^4−^ ligand, which did not have an inhibitory effect on violacein production [87]. Additionally, gallium(III) complexes did not exhibit antagonistic activity on QS in *Serratia marcescens* ATCC 27117, when 100 μg of the tested compound was applied per discs. Overall, these complexes did not modulate violacein biosynthesis in *C. violaceum* CV026 or prodigiosin biosynthesis in *S. marcescens* under the tested conditions.

**Figure 7:**
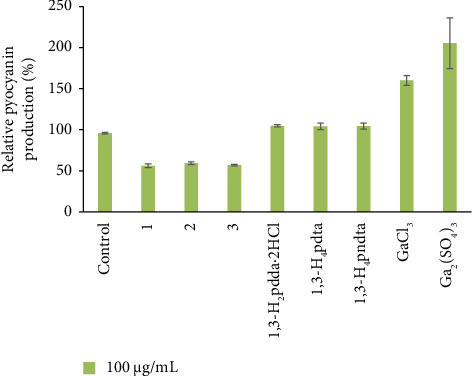
Relative pyocyanin production by *Pseudomonas aeruginosa* BK25H strain in the presence of complexes **1**–**3**, corresponding aminocarboxylic acids, and gallium(III) salts at a concentration of 100 μg/mL.

The effect of Complexes **1**–**3** on *P. aeruginosa* PAO1 biofilm formation was evaluated at subinhibitory concentrations (50% of determined MICs). The results indicated that Complexes **1**–**3** did not inhibit the early stages of biofilm formation. However, these complexes were able to reduce pyocyanin production by 40–43% in the clinical isolate *P. aeruginosa* BK25H (Figure 7 and Table 1), whereas 1,3-H_2_pdda^.^2HCl, 1,3-H_4_pdta, and 1,3-H_4_pndta were not able to interfere with this virulence factor.

In the Gram-negative human pathogen *P. aeruginosa*, there are two chemically distinct yet interconnected QS systems: one dependent on *N*-acyl-(L)-homoserine lactones (AHL) and the other on 2-alkyl-4-quinolones. *P. aeruginosa* produces two AHL molecules, *N*-3-oxo-dodecanoyl-(L)-homoserine lactone (3OC12-HSL) and *N*-butyryl-(L)-homoserine lactone (C4-HSL), which directly or indirectly control the expression of various virulence factors, secondary metabolites, swarming motility, and biofilm development [44, 90]. To assess whether gallium(III) Complexes **1**–**3** influence AHL production in *P. aeruginosa* PAO1, and to differentiate their effects on the rhl and las QS systems, the following specific biosensors were included: PA14-R3ΔlasIPrsaI: lux for the detection of a long-chain AHL (3OC12-HSL) and PAO1 ΔrhlIpKD-rhlA for the detection of a short-chain AHL (C4-HSL). Additionally, the biosensor strain *P. aeruginosa* PAO1 ΔpqsA mini-CTX luxPpqsA was employed to evaluate the impact of Complexes **1**–**3** on 2-alkyl-4-quinolones (AHQs) production in *P. aeruginosa* PAO1.

**Figure 8:**
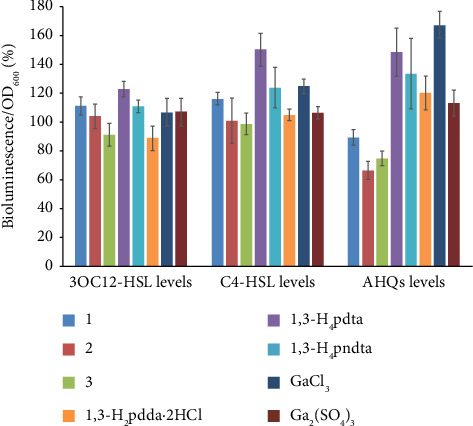
The effect of gallium(III) complexes **1**–**3**, aminocarboxylic acids, and the corresponding gallium(III) salts on acyl homoserine lactone (AHL) and 2-alkyl-4-quinolones (AHQs) production in *P. aeruginosa* PAO1 assessed by various reporter strains. The PA14-R3ΔlasIPrsaI: lux strain was employed to measure 3OC12-HSL levels, the PAO1 ΔrhlIpKD-rhlA strain to measure the C4-HSL levels, and the PAO1ΔpqsA mini-CTX luxPpqsA strain to measure AHQs levels. Values are presented as mean ± SD.

The obtained results showed that Complexes **1** and **3** significantly reduced the production of AHQs (2-alkyl-4-quinolones) by 25% and 33%, respectively, in biosensor strain *P. aeruginosa* PAO1 ΔpqsA mini-CTX luxPpqsA, compared to the untreated control. In contrast, compounds 1,3-H_4_pdta, 1,3-H_4_pndta, and 1,3-H_2_pdda^.^2HCl showed a stimulatory effect of 120–148%, respectively (Figure 8). These results indicate that the investigated gallium(III) complexes with aminocarboxylates can modulate the quinolone-mediated QS system in *P. aeruginosa* PAO1.

Additionally, the cytotoxicity of Complexes **1**–**3**, salts used for their synthesis, and the corresponding aminocarboxylic acids were evaluated against the human fibroblasts cell line MRC-5 (Figure S6). No significant cytotoxic activity of Complexes **1**–**3** was observed in the studied range, indicating that their IC_50_ values are higher than 200 μM. Also, chromium(III) and cobalt(III) complexes with 2,2-diMe-1,3-pdta^4−^ exhibited comparable cytotoxicity profiles, with no cytotoxic effects, even at concentrations of 500 μM [87]. On the other hand, a modest cytotoxicity for 1,3-H_4_pdta and 1,3-H_4_pndta was observed at the highest concentrations (200 and 100 μM, respectively), while GaCl_3_ and Ga_2_(SO_4_)_3_ were safe even at the highest tested concentrations (Figure S6). Similar to our results, gallium(III) complexes with thiosemicarbazones have shown low toxicity to healthy MRC-5 cells, while the effect on the human tumor cell lines (alveolar basal epithelial cells A549, bladder cancer cell line T24, and breast cancer cell line MCF-7) was more pronounced, making them good candidates for the design of selective antitumor agents [91]. The same is true for *tris*(5-nitro-8-quinolinolato)gallium(III) complex, which manifested high antiproliferative effect (IC_50_ = 3.6 μM for colon cancer cell line HCT116) with noticeable selectivity (IC_50_ = 32 μM for normal MRC-5 cells) [92].

### 3.4. Interactions of Complex **1** With Biomolecules

#### 3.4.1. BSA Binding Study and Competitive Experiments With BSA Site Markers

Serum albumin is one of the most abundant plasma proteins and plays an important role in the transport and delivery of different compounds to the target cells [93]. In the present study, the interaction of Complex **1** with BSA, which is structural analog of the human serum albumin, was investigated by fluorescence emission spectroscopy, without and in the presence of site markers (eos Y as a marker for the Site I [subdomain IIA], ibu as a marker for Site II [subdomain IIIA], and dig for Site III [subdomain IB]) [94].

**Figure 9:**
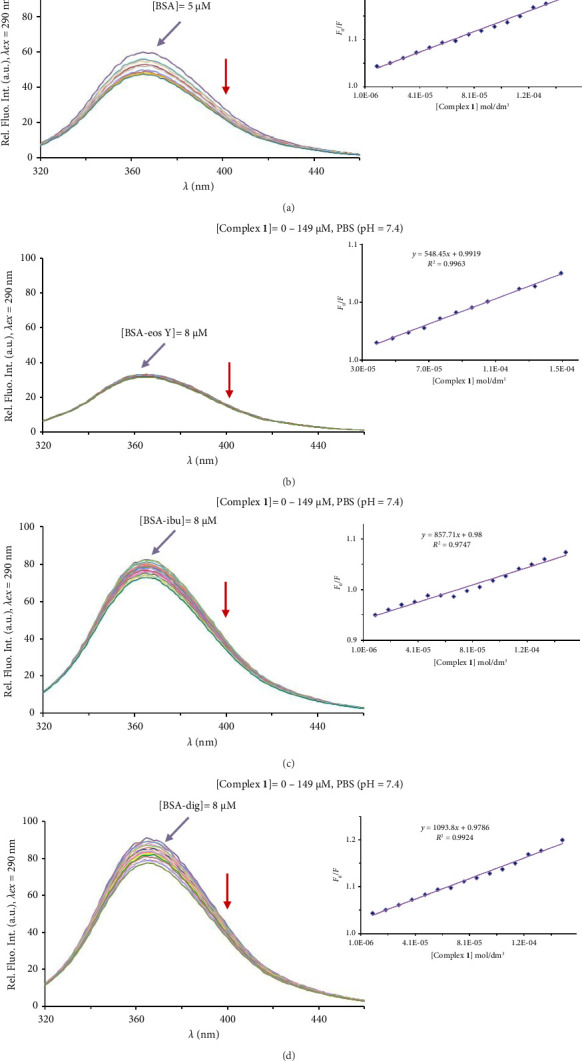
BSA fluorescence emission spectra in the presence of an increasing concentration of Complex **1** (a) and in the presence of the site markers (b–d). The red arrow shows the changes in the intensity after the addition of the complex. The inserted graph presents the F_0_/F dependence from the concentration of complex **1**.

First, the fluorescence emission spectra of BSA were recorded in the presence of an increasing concentration of Complex **1** (Figure 9). As can be noticed, the fluorescence intensity of the studied biomolecule decreases upon increasing the concentration of the complex, as a consequence of their interaction. The obtained fluorescence quenching data are presented in Table 2.

The value of *K*_*Α*_ binding constant for **1** is high enough to indicate its binding to BSA, which can transport this complex to the corresponding biological targets, but not too high to prevent its release from the BSA upon arrival to the target site [95]. The *K*_*Α*_ value for **1** is almost 10-fold higher than those for the Complexes **2** and **3** analyzed in the present study for their antimicrobial potential (vide supra) [34]. The calculated value of the *K*_*q*_ is higher than the maximum diffusion constant of biomolecule (2 × 10^10^ M^−1^s^−1^), indicating the static mechanism of BSA fluorescence quenching [96], while the number of binding sites per BSA molecule suggested that Complex **1** binds to one site per protein.

When the site marker is added to the protein solution, a decrease in fluorescence intensity of BSA can be observed, which indicates that the site marker binds to the BSA. If the complex binds to the same site as the corresponding marker, after addition to the BSA-marker system, it will compete with the marker for BSA, leading to a decrease in the *K*_*A*_ value when compared with that in the absence of the site marker [97]. Based on the results presented in Table 2, the largest decrease of the BSA binding constants of Complex **1** is observable in the presence of eos Y, indicating that it may compete with this site marker and bind to Site I of BSA. However, the similar change in the *K*_*A*_ values for this complex could also be noted in the presence of ibu, and to clarify this, we have decided to perform a molecular docking study.

**Figure 10:**
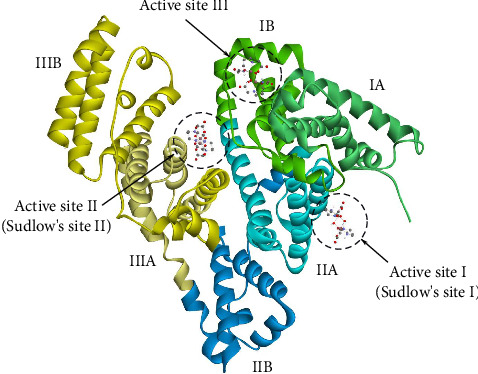
3D representations of the most stable conformations of Complex **1** bound to three distinct active sites (I in subdomain IIA, II in subdomain IIIA, and III in subdomain IB) of BSA (PDB: **4F5S**), with color-coded domains: green for Domain I, blue for Domain II, and yellow for Domain III.

Among the binding sites of BSA are Site I, situated in subdomain IIA, Site II, in subdomain IIIA, and Site III, near the subdomain interface IB. These sites are essential for the coordination of several endogenous and external ligands [98]. In Figure 10, the spatial configuration and accommodation of Complex **1** within these active sites are illustrated.

The results obtained by molecular docking indicate varying affinities of Complexes **1**–**3** for the three recognized binding sites on BSA (Table 3). Complex **1** demonstrated the strongest interaction with binding Site I, with a binding energy (ΔG_bind_) of −9.77 kcal/mol^−1^, suggesting a potentially high-affinity interaction at this site. With ΔG_bind_ of −7.22 and −5.37 kcal/mol, respectively, its affinity for Sites II and III was noticeably lower. Complex **2** exhibited moderate binding values across all three sites, while Complex **3** displayed a relatively balanced binding profile, with differences in ΔG_bind_ across the sites of less than 1 kcal/mol.

The results of molecular docking support experimental observations, since Complex **1** had the most robust binding to Site I, but its interaction with Site II was comparatively weaker but still significant. Complexes **2** and **3** exhibited a reduced degree of selectivity toward any one site, as seen by their rather consistent binding energies across all three sites. The results indicate that Complex **1** exhibits the highest selectivity toward Site I, whereas Complexes **2** and **3** may have more extensive scopes of action, engaging with several binding sites on BSA.

**Figure 11:**
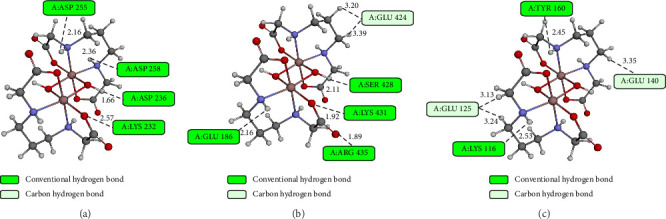
2D representation of the interactions between complex **1** and BSA with interatomic distances (Å) obtained from the molecular docking study in different active sites: I (a), II (b), and III (c). Different colors indicate the types of interactions (see legend). Legend: Ga—pink, C—grey, H—white, O—red, and N—blue.

The interactions between the investigated complexes and amino acids located inside the binding sites of BSA are illustrated in Figures 11, S7, and S8. Conventional hydrogen bonds are the prevailing type of bonds formed between Complex **1** and the amino acids in all three active sites. The observed interaction indicates that Complex **1** establishes stable hydrogen bonds with crucial residues within the binding pockets, therefore enhancing its binding affinity, notably at Site I.

Conversely, Complexes **2** and **3** predominantly participate in attractive charge interactions with the amino acids located in the active binding sites (Figures S7 and S8). The observed phenomenon can be attributed to the inherent charge of these complexes, which facilitates electrostatic interactions with the charged residues in BSA. Despite their significance, these charge-based interactions may lead to reduced selectivity and lesser binding energies in comparison with the hydrogen bonding interactions shown with Complex **1**.

The different interaction characteristics seen for the three gallium(III) complexes emphasize their disparate binding processes. Complex **1** demonstrates more robust site-specific hydrogen bonding, whereas Complexes **2** and **3** depend more on charge-based interactions. The observed variation in binding behavior corresponds to the findings from the docking analysis. Complex **1** exhibited the greatest binding affinity toward Site I, while Complexes **2** and **3** had more consistent, albeit less strong, interactions across all three sites.

#### 3.4.2. DNA Binding Study

**Figure 12:**
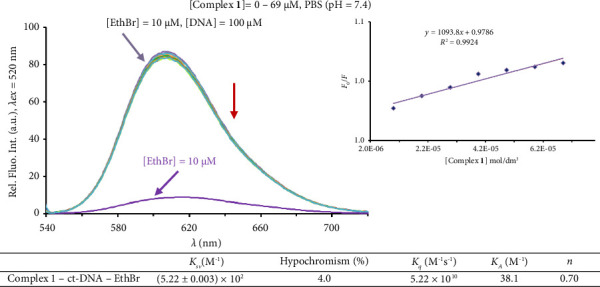
Fluorescence emission spectra of EthBr-ct-DNA system in the presence of an increasing amount of Complex **1** with values of its binding constants. The red arrow shows the intensity changes upon increased concentration of the complex. Inserted graph: Stern–Volmer plot of *F*_0_/*F* vs. [Complex **1**].

The investigation of interactions of transition metal complexes with DNA is of great importance for understanding the molecular mechanism of action and for the design of new DNA-targeted drugs [99]. Accordingly, the binding affinity of Complex **1** toward ct-DNA was investigated by competitive studies with ethidium bromide (EthBr) as an intercalator [47] and 2′-(4-hydroxyphenyl)-5-[5-(4-methylpiperazine-1-yl)benzimidazo-2-yl]-benzimidazole (Hoechst 33258; Hoe) as a minor groove binder [100] using fluorescence emission spectroscopy. The emission spectra of the EthBr-ct-DNA and Hoe-ct-DNA systems were recorded in the presence of an increasing amount of the Complex **1**. Based on the obtained data, no decrease in the fluorescence intensity of Hoe-ct-DNA system was noticed, suggesting no affinity of the complex toward a minor groove. Also, as can be seen in Figure 12, upon the addition of Complex **1**, no significant decrease in the fluorescence intensity of EthBr-ct-DNA system was observed. The calculated *K*_*A*_ constant in Figure 12 for Complex **1** is much lower than that for EthBr (*K*_*A*_ = 2 × 10^6^ M^−1^), confirming the non-intercalative mode of the complex binding [96]. According to the results obtained, it can be concluded that no significant affinity of Complex **1** toward ct-DNA was observed. The low affinity of the Complex **1** toward ct-DNA is in accordance with that of the gallium(III) Complexes **2** and **3** [34].

Analysis of molecular docking results between Complexes **1**–**3** and two DNA conformations (6-bp-DNA and 10-bp-DNA) reveal their specific binding mechanisms with DNA. In the case of 6-bp-DNA, associated with an intercalative binding mode, the binding energy of the tested complexes indicates moderate affinity toward this type of interaction. Complex **1** had the lowest binding energy of −6.88 kcal/mol, indicating its greater capacity for intercalation in comparison with Complexes **2** and **3**, which displayed binding energies of −4.57 and −5.06 kcal/mol, respectively (Table 4). These data suggest that Complexes **2** and **3** are less effective in intercalating with 6-bp-DNA, likely due to their molecular structure and possible steric hindrance that limits their ability to insert between DNA bases.

On the other hand, the docking results with 10-bp-DNA, which accommodates minor groove binding, reveal significantly lower binding energies, especially for Complex **1** (−10.89 kcal/mol). Significant affinity toward the minor groove is shown by Complexes **2** and **3**, with binding energies of −7.85 and −7.99 kcal/mol, respectively (Table 4). These results suggest that Complex **1** has a much greater propensity for binding to the minor groove of DNA compared to intercalative sites, potentially due to its structure and ability to form hydrogen bonds or other interactions with the DNA bases within the minor groove. Although Complexes **2** and **3** have a lesser affinity for the minor groove than Complex **1**, their affinity remains considerably greater than their intercalative binding.

The observed discrepancy between theoretical predictions and experimental results can be attributed to several factors. Simulations, including molecular docking, frequently do not correctly represent the complete spectrum of physiological conditions in experimental systems, such as solvation effects, ionic strength, or the distinct conformational dynamics of both DNA and the complex in solution. In docking studies, the structures of DNA and complexes are frequently treated as rigid, whereas in reality, their flexibility and dynamic behavior play a crucial role in their interaction. Moreover, experimental parameters such as DNA concentration, possible molecular aggregation, and extrinsic factors (e.g., pH, co-ions) can substantially alter the interaction. These variations can lead to a lower binding affinity observed in experimental measurements than what is predicted by theoretical models. These results highlight the necessity of combining experimental and theoretical methodologies to attain a more thorough comprehension of the interaction processes of these complexes with DNA.

**Figure 13:**
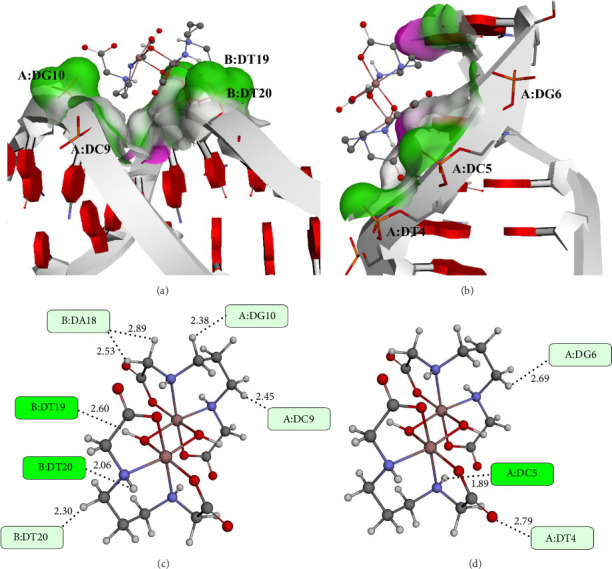
Three-dimensional representations show the most stable conformations of Complex **1** as intercalator in (a) 10-bp-DNA (PDB: **1BNA**) and minor groove binder in (b) 6-bp-DNA (PDB: **1Z3F**), with the sugar-phosphate backbones depicted as helically twisted white bands and nucleobases in blue. Figures (c) and (d) highlight the interactions between Complex **1** and both DNA sequences, showing interatomic distances (Å) from docking simulations. Nucleotides are labeled (DA = deoxyadenosine; DG = deoxyguanosine; DC = deoxycytidine; DT = deoxythymidine), with interaction types color-coded. The investigated compounds are shown as grey carbon sticks, and atoms are represented by spheres: N (blue), O (red), H (white), and Ga (pink).

Molecular docking studies reveal specific interactions between the investigated compounds and nucleotides within DNA chains, as shown in Figures 13, S9, and S10. The investigation complexes are mainly stabilized in the 10-bp-DNA conformation by conventional hydrogen bonding with nucleotides A:DG4, B:DT19, B:DT20, and B:DG22. Furthermore, the studied compounds exhibit carbon–hydrogen bonding with nucleotides A:DA6, A:DC9, A:DG10, B:DA18, and B:DT20. These interactions contribute significantly to the stabilization of the complexes within the minor groove of 10-bp-DNA, reflecting their strong binding affinity.

In contrast, interactions between the investigated compounds and 6-bp-DNA are characterized by fewer conventional hydrogen bonds, specifically with nucleotides DC5, DG2, and DG6. This limited number of hydrogen bonds indicates weaker stabilization of the compounds in 6-bp-DNA, which may account for the lower binding affinities compared to 10-bp-DNA.

Additionally, Complexes **2** and **3**, due to their anionic nature, establish characteristic electrostatic interactions, including attractive charge interactions with nucleotide B:DG2. These electrostatic interactions provide additional stabilization for Complexes **2** and **3**, compensating for the limited hydrogen bonding and enhancing their binding to DNA.

The results obtained from the examination of the interactions of Complex **1** with biomolecules significantly contribute to understanding its potential pharmacological profile and biological compatibility. The identification of specific binding sites on BSA and the nature of these interactions indicates the possibility of selective transport of this complex to targeted biological sites. These investigations establish a basis for an enhanced understanding of the mechanisms of action and interactions between metal complexes and essential biomolecules, paving the way for their advancement in therapeutic applications. Furthermore, the results show that, although Complex **1** does not exhibit significant affinity for DNA, it has a favorable safety profile, making it potentially suitable for further investigations in biomedical applications. These findings highlight the broader importance of chemical stability, protein interactions, and binding selectivity for applications in bioinorganic chemistry and drug development.

## 4. Conclusions

In this study, a dinuclear gallium(III) complex, uns-*cis*-[Ga(1,3-pdda)(*µ*-OH)]_2_·2H_2_O (**1**), with tetradentate 1,3-propanediamine-*N,N′*-diacetate (1,3-pdda^2-^) ligand was successfully synthesized, and its structure was confirmed using IR and NMR spectroscopy, as well as single-crystal X-ray diffraction analysis. The structure of Complex **1** was further validated using DFT methods, with a high correlation factor between experimental and theoretical values of structural parameters, indicating that the chosen theoretical level accurately describes the molecular structure. HSA highlighted the significance of intermolecular interactions, particularly H⋯H and H⋯O/O⋯H bonds, for the crystal stability. The antimicrobial activity of Complex **1** was compared with that of gallium(III) complexes previously characterized, Na[Ga(1,3-pdta)]·3H_2_O (**2**) and Ba[Ga(1,3-pndta)]_2_·3H_2_O (**3**), and interactions of **1** with ct-DNA and BSA protein was also investigated. Results indicated that these gallium(III) complexes manifested selective antibacterial activity against *Pseudomonas aeruginosa* PAO1 strain and modulated the quinolone-mediated QS system in this bacterium. Spectrofluorimetric experiments and molecular docking revealed that Complex **1** binds strongly to Site I on BSA, whereas Complexes **2** and **3** showed less selective binding. Although spectrofluorimetric results and docking studies indicated weak affinity of Complex **1** for ct-DNA, docking simulations suggested potential binding to the minor groove, particularly in larger DNA sequences. These findings provide valuable insights into molecular interactions and offer new directions for further research into the coordination and medicinal chemistry of gallium(III) complexes.

## Figures and Tables

**Table 1:** Antimicrobial activity of the investigated complexes **1**–**3**, aminocarboxylic acids, and the corresponding gallium(III) salts used for their synthesis (MIC, μM) against *P. aeruginosa* PAO1 in comparison with their inhibitory effect on pyocyanin production in *P. aeruginosa* BK25H.

	1	2	3	1,3-H_2_pdda·2HCl	1,3-H_4_pdta	1,3-H_4_pndta	GaCl_3_	Ga_2_(SO_4_)_3_
*P. aeruginosa* (MIC, μM)	31.25	125	125	> 500	> 500	> 500	31.25	15.625
Pyocyanin inhibition (%)	44	40	43	/	/	/	/	/

*Note:* Subinhibitory concentration for pyocyanin assessment was 100 μg/mL.

**Table 2:** Values of the BSA binding data for complex **1** in the absence and presence of the site markers, eosin Y (eos Y), ibuprofen (ibu), and digitoxin (dig).

	*K*_*sv*_ (M^−1^)	Hypochromism (%)	*K*_*q*_ (M^−1^s^−1^)	*K*_*A*_ (M^−1^)	*n*
1—BSA	(2.79 ± 0.008) × 10^3^	20.3	2.79 × 10^11^	1.16 × 10^3^	0.90
1—BSA—eos Y	(5.53 ± 0.001) × 10^2^	7.9	5.53 × 10^10^	3.32 × 10^2^	0.94
1—BSA—ibu	(8.75 ± 0.003) × 10^2^	11.9	8.75 × 10^10^	4.91 × 10^2^	0.94
1—BSA—dig	(1.12 ± 0.002) × 10^3^	14.6	1.12 × 10^11^	9.37 × 10^2^	0.99

**Table 3:** Thermodynamic parameters (Δ*G*_bind_ binding free energy, *K*_*i*_ inhibition constant, Δ*G*_total_ total energy, Δ*G*_tor_ torsional free energy, Δ*G*_unb_ unbound system energy, Δ*G*_elec_ electrostatic energy, and Δ*G*_vdw+hbond+desolv_ as the sum of van der Waals, hydrogen bond, and desolvation energies) for the most favorable conformations of complexes **1**–**3** in BSA active sites.

Active site	Δ*G*_bind_	*K*_*i*_ (µM)	Δ*G*_inter_	Δ*G*_vdw+hbond+desolv_	Δ*G*_elec_	Δ*G*_total_	Δ*G*_tor_	Δ*G*_unb_
**1**								
I	−9.77	0.68	−9.77	−7.89	−1.89	0.00	0.00	0.00
II	−7.22	5.08	−7.22	−4.32	−2.91	0.00	0.00	0.00
III	−5.37	115.2	−5.37	−6.73	1.36	0.00	0.00	0.00
**2**								
I	−6.64	13.65	−6.64	−5.25	−1.39	0.00	0.00	0.00
II	−5.74	62.36	−5.74	−5.16	−0.58	0.00	0.00	0.00
III	−5.45	100.4	−5.45	−3.91	−1.51	0.00	0.00	0.00
**3**								
I	−6.38	21.01	−6.66	−5.08	−1.58	−0.13	0.27	−0.13
II	−6.13	32.12	−6.40	−5.50	−0.91	0.67	0.27	0.67
III	−6.00	40.20	−6.27	−4.50	−1.77	−0.16	0.27	−0.16

**Table 4:** Thermodynamic parameters (Δ*G*_bind_ binding free energy, *K*_*i*_ inhibition constant, Δ*G*_total_ total energy, Δ*G*_tor_ torsional free energy, Δ*G*_unb_ unbound system energy, Δ*G*_elec_ electrostatic energy, and Δ*G*_vdw+hbond+desolv_ as the sum of van der Waals, hydrogen bond, and desolvation energies) for the most favorable conformations of complexes **1**–**3** in DNA conformations (6-bp-DNA and 10-bp-DNA).

Comp.	Δ*G*_bind_	*K*_*i*_ (µM)	Δ*G*_inter_	Δ*G*_vdw+hbond+desolv_	Δ*G*_elec_	Δ*G*_total_	Δ*G*_tor_	Δ*G*_unb_
6-bp-DNA								
**1**	−6.88	9.09	−6.88	−3.82	−3.05	0.00	0.00	0.00
**2**	−4.57	445.7	−4.57	−4.45	−0.12	0.00	0.00	0.00
**3**	−5.06	194.7	−5.34	−4.92	−0.42	−0.15	0.27	−0.15
10-bp-DNA								
**1**	−10.89	0.10	−10.89	−4.34	−6.55	0.00	0.00	0.00
**2**	−7.85	1.76	−7.85	−6.70	−1.15	0.00	0.00	0.00
**3**	−7.99	1.39	−8.27	−7.39	−0.88	−0.15	0.27	−0.15

## Data Availability

The data that support the findings of this study are available from the corresponding author upon reasonable request.

## Supporting Information

Supporting Information associated with this article includes Tables S1–S7 and Figures S1–S10. Table S1: Details of the crystal structure determination for Complex **1**; Table S2: Selected experimental and theoretical bond distances (Å) and bond angles (°) for Complex **1**; Table S3: Hydrogen bonding interactions in Complex **1**; Table S4: Cremer and Pople puckering parameters [69] of the five- and six-membered chelate rings in uns*-cis*-[Ga(1,3-pdda)(µ-OH)]_2_^.^2H_2_O (**1**) and uns*-cis*-[Co(1,3-pdda)(µ-H_2_O)]_2_·4H_2_O complexes; Table S5: Analysis of octahedral distortion in dinuclear six-coordinate 1,3-pdda^2−^ complexes of gallium(III) and cobalt(II) of uns*-cis* configuration; Table S6: The IR positions of asymmetric (*ν*_*asym*_) and symmetric (*ν*_*sym*_) stretching frequencies (cm^−1^) of carboxyl groups of 1,3-H_2_pdda·2HCl in comparison with those for the coordinated carboxylate groups in Complexes **1**–**3**; Table S7: The experimental and calculated chemical shifts (*δ*, ppm) in the ^1^H and ^13^C NMR spectra of Complex **1**; Figure S1: The structural formula of ethylenediamine-*N,N′*-diacetate (edda^2−^) ligand (A) and possible geometrical isomers for the octahedral metal complexes containing tetradentate edda^2−^ and two monodentate (X) ligands (B); Figure S2: ^1^H NMR spectrum of Complex **1** in D_2_O at 600 MHz at room temperature; Figure S3: ^13^C NMR spectrum of Complex **1** in D_2_O at 150 MHz at room temperature; Figure S4: ^1^H NMR spectra of Complex **1** recorded immediately and 48 h after dissolution in D_2_O at room temperature (600 MHz); Figure S5: Inhibition of violacein production in the presence of Complexes **1**–**3**, salts used for their synthesis (GaCl_3_ and Ga_2_(SO_4_)_3_) and corresponding aminocarboxylic acids on *Chromobacterium violaceum* CV026 at 100 μg/disc. H_2_O was used as a control; Figure S6: Cytotoxic effect of Complexes **1**–**3**, corresponding aminocarboxylic acids and gallium(III) salts on the MRC-5 cells. Values are presented as mean ± SD; Figure S7: 2D representation of the interactions between Complex **2** and BSA with interatomic distances (Å) obtained from the molecular docking study in different active sites: I (A), II (B), and III (C). Different colors indicate the types of interactions (see legend). Legend: Ga—pink, C—gray, H—white, O—red, N—blue; Figure S8: 2D representation of the interactions between Complex **3** and BSA with interatomic distances (Å) obtained from the molecular docking study in different active sites: I (A), II (B), and III (C). Different colors indicate the types of interactions (see legend). Legend: Ga—pink, C—grey, H—white, O—red, N—blue; Figure S9: Three-dimensional representations show the most stable conformations of Complex **2** as intercalator in (A) 10-bp-DNA (PDB: **1BNA**) and minor groove binder in (B) 6-bp-DNA (PDB: **1Z3F**), with the sugar-phosphate backbones depicted as helically twisted white bands and nucleobases in blue. Figures (C) and (B) highlight the interactions between Complex **2** and both DNA sequences, showing interatomic distances (Å) from docking simulations. Nucleotides are labeled (DA = deoxyadenosine; DG = deoxyguanosine; DC = deoxycytidine; DT = deoxythymidine), with interaction types color-coded. The investigated compounds are shown as grey carbon sticks, and atoms are represented by spheres: N (blue), O (red), H (white), and Ga (pink); Figure S10: Three-dimensional representations show the most stable conformations of Complex **3** as intercalator in (A) 10-bp-DNA (PDB: **1BNA**) and minor groove binder in (B) 6-bp-DNA (PDB: **1Z3F**), with the sugar-phosphate backbones depicted as helically twisted white bands and nucleobases in blue. Figures (C) and (B) highlight the interactions between Complex **3** and both DNA sequences, showing interatomic distances (Å) from docking simulations. Nucleotides are labeled (DA = deoxyadenosine; DG = deoxyguanosine; DC = deoxycytidine; DT = deoxythymidine), with interaction types color-coded. The investigated compounds are shown as grey carbon sticks, and atoms are represented by spheres: N (blue), O (red), H (white), and Ga (pink).

Cif and CheckCif files for Complex **1** are provided under the Supporting section. CCDC 2380346 contains the supporting crystallographic data for this paper. These data can be obtained free of charge from the Cambridge Crystallographic Data Center via https://www.ccdc.cam.ac.uk/data_request/cif.
