# Supplementary material for: Dinuclear Gallium(III) Complex With 1,3-Propanediamine-N,N′-Diacetate: Structural Characterization, Antimicrobial Activity, and DNA/BSA Interactions
Source: Bioinorg Chem Appl. 2025 Apr 12;2025:8097589. doi: 10.1155/bca/8097589 (PMC12011468; doi:10.1155/bca/8097589)

checkCIF/PLATON report

Structure factors have been supplied for datablock(s) 1

THIS REPORT IS FOR GUIDANCE ONLY. IF USED AS PART OF A REVIEW PROCEDURE FOR PUBLICATION, IT SHOULD NOT REPLACE THE EXPERTISE OF AN EXPERIENCED CRYSTALLOGRAPHIC REFEREE.

No syntax errors found. CIF dictionary Interpreting this report

Datablock: 1

```
Bond precision:   C-C = 0.0050 A          Wavelength=0.71073

Cell:             a=7.7399(6)     b=7.8024(5)        c=17.8058(13)
                  alpha=90        beta=98.937(7)     gamma=90
Temperature:      150 K

                Calculated                  Reported
Volume          1062.24(13)                 1062.24(13)
Space group     P 21/c                      P 21/c
Hall group      -P 2ybc                     -P 2ybc
Moiety formula  C14 H26 Ga2 N4 O10, 2(H2 O) C14 H26 Ga2 N4 O10, 2(H2 O)
Sum formula     C14 H30 Ga2 N4 O12          C14 H30 Ga2 N4 O12
Mr              585.86                      585.86
Dx,g cm-3       1.832                       1.832
Z               2                           2
Mu (mm-1)       2.607                       2.607
F000            600.0                       600.0
F000'           601.18
h,k,lmax        10,10,23                    10,10,23
Nref            2435                        2434
Tmin,Tmax       0.803,0.878                 0.658,1.000
Tmin'           0.594

Correction method= # Reported T Limits: Tmin=0.658 Tmax=1.000
AbsCorr = MULTI-SCAN

Data completeness= 1.000          Theta(max)= 27.483

                                                     wR2(reflections)=
R(reflections)= 0.0409( 1944)                        0.1000( 2434)

S = 1.108                 Npar= 160
```

The following ALERTS were generated. Each ALERT has the format
test-name_ALERT_alert-type_alert-level.
Click on the hyperlinks for more details of the test.

Alert level C

```
PLAT411_ALERT_2_C Short Inter H...H Contact  H3B      ..H3B      .       2.14 Ang.
                                          -x,-y,2-z  =      3_557 Check
PLAT975_ALERT_2_C Check Calcd Resid. Dens.  0.97Ang From O2      .       0.50 eA-3
PLAT975_ALERT_2_C Check Calcd Resid. Dens.  0.42Ang From O5      .       0.41 eA-3
```

Alert level G

```
PLAT002_ALERT_2_G Number of Distance or Angle Restraints on AtSite          9 Note
PLAT172_ALERT_4_G The CIF-Embedded .res File Contains DFIX Records          3 Report
PLAT793_ALERT_4_G Model has Chirality at N1          (Centro SpGr)          R Verify
PLAT793_ALERT_4_G Model has Chirality at N2          (Centro SpGr)          R Verify
PLAT860_ALERT_3_G Number of Least-Squares Restraints ............           5 Note
PLAT899_ALERT_4_G SHELXL2018 is Outdated   and Succeeded by SHELXL     2019/3 Note
PLAT910_ALERT_3_G Missing # of FCF Reflection(s) Below Theta(Min).          1 Note
                0  0  2,
PLAT941_ALERT_3_G Average HKL Measurement Multiplicity ..........         3.1 Low
PLAT955_ALERT_1_G Reported (CIF) and Actual (FCF) Lmax Differ by .          1 Units
PLAT969_ALERT_5_G The 'Henn et al.' R-Factor-gap value ..........       2.680 Note
              Predicted wR2: Based on SigI**2  3.73 or SHELX Weight  9.03
PLAT978_ALERT_2_G Number C-C Bonds with Positive Residual Density.          1 Info
```

0 **ALERT level A** = Most likely a serious problem - resolve or explain
0 **ALERT level B** = A potentially serious problem, consider carefully
3 **ALERT level C** = Check. Ensure it is not caused by an omission or oversight
11 **ALERT level G** = General information/check it is not something unexpected

1 ALERT type 1 CIF construction/syntax error, inconsistent or missing data
5 ALERT type 2 Indicator that the structure model may be wrong or deficient
3 ALERT type 3 Indicator that the structure quality may be low
4 ALERT type 4 Improvement, methodology, query or suggestion
1 ALERT type 5 Informative message, check

checkCIF publication errors

Alert level A

```
PUBL006_ALERT_1_A  _publ_requested_journal is missing
            e.g. 'Acta Crystallographica Section C'
PUBL008_ALERT_1_A  _publ_section_title is missing. Title of paper.
PUBL012_ALERT_1_A  _publ_section_abstract is missing.
            Abstract of paper in English.
```

Alert level G

```
PUBL017_ALERT_1_G The _publ_section_references section is missing or
            empty.
```

```
   3 ALERT level A = Data missing that is essential or data in wrong format
   1 ALERT level G = General alerts. Data that may be required is missing
```

Publication of your CIF

You should attempt to resolve as many as possible of the alerts in all categories. Often the minor alerts point to easily fixed oversights, errors and omissions in your CIF or refinement strategy, so attention to these fine details can be worthwhile. In order to resolve some of the more serious problems it may be necessary to carry out additional measurements or structure refinements. However, the nature of your study may justify the reported deviations from journal submission requirements and the more serious of these should be commented upon in the discussion or experimental section of a paper or in the "special_details" fields of the CIF. *checkCIF* was carefully designed to identify outliers and unusual parameters, but every test has its limitations and alerts that are not important in a particular case may appear. Conversely, the absence of alerts does not guarantee there are no aspects of the results needing attention. It is up to the individual to critically assess their own results and, if necessary, seek expert advice.

If level A alerts remain, which you believe to be justified deviations, and you intend to submit this CIF for publication in a journal, you should additionally insert an explanation in your CIF using the Validation Reply Form (VRF) below. This will allow your explanation to be considered as part of the review process.

Validation response form

Please find below a validation response form (VRF) that can be filled in and pasted into your CIF.

```
# start Validation Reply Form
_vrf_PUBL006_GLOBAL
;
PROBLEM: _publ_requested_journal is missing
RESPONSE: ...
;
_vrf_PUBL008_GLOBAL
;
PROBLEM: _publ_section_title is missing. Title of paper.
RESPONSE: ...
;
_vrf_PUBL012_GLOBAL
;
PROBLEM: _publ_section_abstract is missing.
RESPONSE: ...
;
# end Validation Reply Form
```

If you wish to submit your CIF for publication in Acta Crystallographica Section C or E, you should upload your CIF via the web. If you wish to submit your CIF for publication in IUCrData you should upload your CIF via the web. If your CIF is to form part of a submission to another IUCr journal, you will be asked, either during electronic submission or by the Co-editor handling your paper, to upload your CIF via our web site.

PLATON version of 22/08/2024; check.def file version of 21/08/2024

Datablock 1 - ellipsoid plot

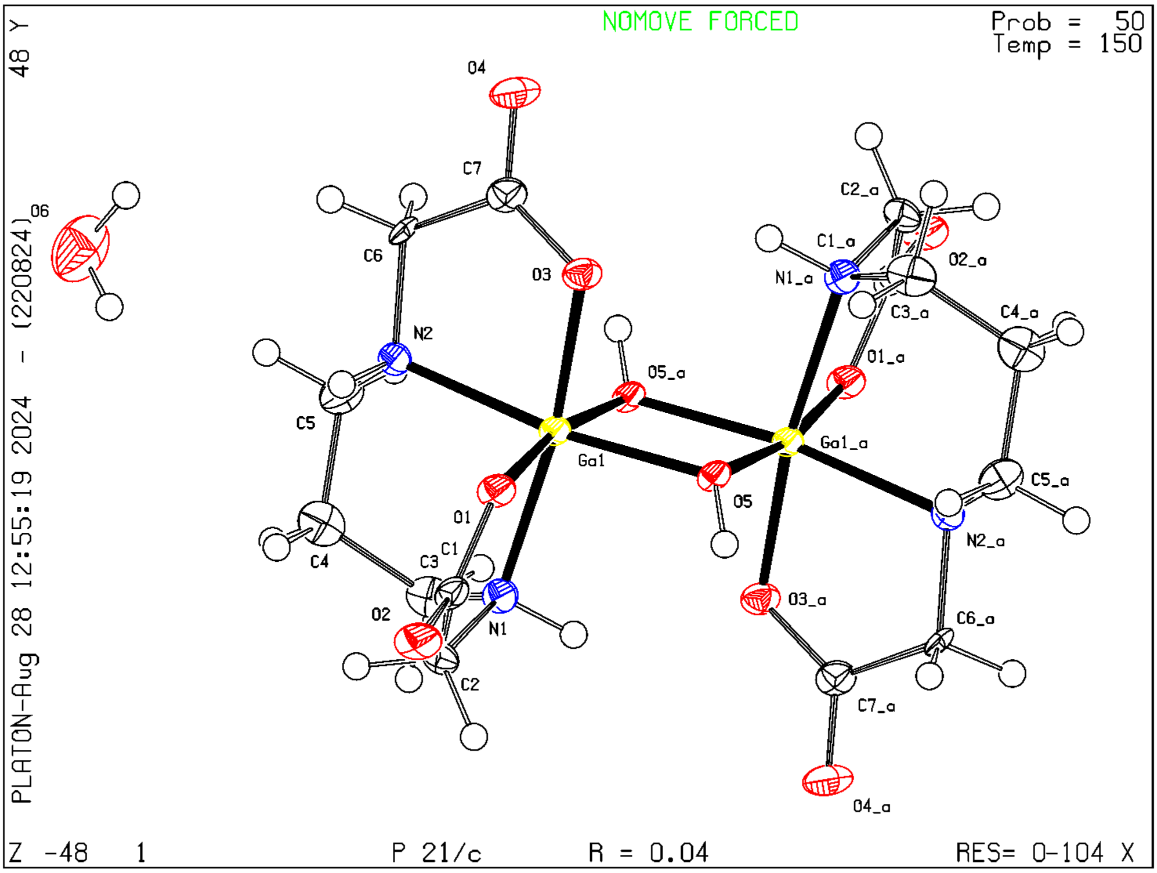

Supplement: Supporting Information — Additional supporting information can be found online in the Supporting Information section. [file 8097589.f1.zip › structure1-checkcif.pdf]
