# Supplementary material for: Dinuclear Gallium(III) Complex With 1,3-Propanediamine-N,N′-Diacetate: Structural Characterization, Antimicrobial Activity, and DNA/BSA Interactions
Source: Bioinorg Chem Appl. 2025 Apr 12;2025:8097589. doi: 10.1155/bca/8097589 (PMC12011468; doi:10.1155/bca/8097589)
Supplement: Supporting Information — Additional supporting information can be found online in the Supporting Information section. [file 8097589.f1.zip › Supplementary Material_8097589_revised.docx]

**Dinuclear Gallium(III) Complex with 1,3-Propanediamine-*N,N’*-Diacetate: Structural Characterization, Antimicrobial Activity and DNA/BSA Interactions**

**TABLE OF CONTENTS**

| Table S1: Details of the crystal structure determination for complex **1**. | S3 |
| --- | --- |
| Table S2: Selected experimental and theoretical bond distances (Å) and bond angles (°) for complex **1**. | S4 |
| Table S3: Hydrogen bonding interactions in complex **1**. | S5 |
| Table S4: Cremer and Pople puckering parameters [69] of the five- and six-membered chelate rings in uns*-cis*-[Ga(1,3-pdda)(*µ*-OH)]_2_^.^2H_2_O (**1**) and uns*-cis*-[Co(1,3-pdda)(*µ*-H_2_O)]_2_^.^4H_2_O complexes. | S6 |
| Table S5: Analysis of octahedral distortion in dinuclear six-coordinate 1,3-pdda^2−^ complexes of gallium(III) and cobalt(II) of uns*-cis* configuration. | S7 |
| Table S6: The IR positions of asymmetric (*ν*_asym_) and symmetric (*ν*_sym_) stretching frequencies (cm^-1^) of carboxyl groups of 1,3-H_2_pdda^.^2HCl in comparison to those for the coordinated carboxylate groups in complexes **1** – **3**. | S8 |
| Table S7: The experimental and calculated chemical shifts (*δ*, ppm) in the ^1^H and ^13^C NMR spectra of complex **1**. | S9 |
| Figure S1: The structural formula of ethylenediamine-*N,N’*-diacetate (edda^2˗^) ligand (A) and possible geometrical isomers for the octahedral metal complexes containing tetradentate edda^2-^ and two monodentate (X) ligands (B). | S10 |
| Figure S2: ^1^H NMR spectrum of complex **1** in D_2_O at 600 MHz at room temperature. | S11 |
| Figure S3: ^13^C NMR spectrum of complex **1** in D_2_O at 150 MHz at room temperature. | S12 |
| Figure S4: ^1^H NMR spectra of complex **1** recorded immediately and 48 h after dissolution in D_2_O at room temperature (600 MHz). | S13 |
| Figure S5: Inhibition of violacein production in the presence of complexes **1** – **3**, salts used for their synthesis (GaCl_3_ and Ga_2_(SO_4_)_3_) and corresponding aminocarboxylic acids on *Chromobacterium* *violaceum* CV026 at 100 µg/disc. H_2_O was used as a control. | S14 |
| Figure S6: Cytotoxic effect of complexes **1** – **3**, corresponding aminocarboxylic acids and gallium(III) salts on the MRC-5 cells. Values are presented as mean ± SD. | S15 |
| Figure S7: 2D representation of the interactions between complex **2** and BSA with interatomic distances (Å) obtained from the molecular docking study in different active sites: I (A), II (B), and III (C). Different colours indicate the types of interactions (see legend). Legend: Ga – pink, C – grey, H – white, O – red, N – blue. | S16 |
| Figure S8: 2D representation of the interactions between complex **3** and BSA with interatomic distances (Å) obtained from the molecular docking study in different active sites: I (A), II (B), and III (C). Different colours indicate the types of interactions (see legend). Legend: Ga – pink, C – grey, H – white, O – red, N – blue. | S17 |
| Figure S9: Three-dimensional representations show the most stable conformations of complex **2** as intercalator in (A) 10-bp-DNA (PDB: **1BNA**) and minor groove binder in (B) 6-bp-DNA (PDB: **1Z3F**), with the sugar-phosphate backbones depicted as helically twisted white bands and nucleobases in blue. Figures (C) and (B) highlight the interactions between complex **2** and both DNA sequences, showing interatomic distances (Å) from docking simulations. Nucleotides are labelled (DA = deoxyadenosine; DG = deoxyguanosine; DC = deoxycytidine; DT = deoxythymidine), with interaction types colour-coded. The investigated compounds are shown as grey carbon sticks, and atoms are represented by spheres: N (blue), O (red), H (white) and Ga (pink). | S18 |
| Figure S10: Three-dimensional representations show the most stable conformations of complex **3** as intercalator in (A) 10-bp-DNA (PDB: **1BNA**) and minor groove binder in (B) 6-bp-DNA (PDB: **1Z3F**), with the sugar-phosphate backbones depicted as helically twisted white bands and nucleobases in blue. Figures (C) and (B) highlight the interactions between complex **3** and both DNA sequences, showing interatomic distances (Å) from docking simulations. Nucleotides are labelled (DA = deoxyadenosine; DG = deoxyguanosine; DC = deoxycytidine; DT = deoxythymidine), with interaction types colour-coded. The investigated compounds are shown as grey carbon sticks, and atoms are represented by spheres: N (blue), O (red), H (white) and Ga (pink). | S19 |

Table S1: Details of the crystal structure determination for complex **1**.

| **1** | |
| --- | --- |
| CCDC number | 2380346 |
| Formula | C_14_H_30_Ga_2_N_4_O_12_ |
| *M*_r_ | 585.86 |
| *T* (K) | 150.00(10) |
| Crystal system | monoclinic |
| Space group | *P*2_1_/*c* |
| *a* (Å) | 7.7399(6) |
| *b* (Å) | 7.8024(5) |
| *c* (Å) | 17.8058(13) |
| *α* (°) | 90 |
| *β* (°) | 98.937(7) |
| *γ* (°) | 90 |
| Volume (Å^3^) | 1062.24(13) |
| Z | 2 |
| *D*_c_ (g/cm^3^) | 1.832 |
| *μ* (mm^–1^) | 2.607 |
| *F*(000) | 600.0 |
| Reflections collected | 7438 |
| *R*_int_ | 0.0426 |
| Data/restraints/parameters | 2434/5/160 |
| *R*, *wR*_2_ [*I* > 2σ(*I*)] *^a^* | 0.0409, 0.0895 |
| *R*, *wR*_2_ (all data) *^a^* | 0.0409, 0.0895 |
| GOF, *S* ^b^ | 1.108 |
| Largest diff. peak/hole / e/Å^3^ | 0.78/–0.88 |

*^a^* *R* = ∑||*F*_o_| – |*F*_c_||/∑|*F*_o_|, *wR*_2_ = {∑[*w*(*F*_o_^2^ – *F*_c_^2^)^2^]/∑[*w*(*F*_o_^2^)^2^]}^1/2^.
*^b^* *S* = {∑[(*F*_o_^2^ – *F*_c_^2^)^2^]/(*n* – *p*)}^1/2^, where *n* is the number of reflections and *p* is the total number of refined parameters.

Table S2: Selected experimental and theoretical bond distances (Å) and bond angles (°) for complex **1**.

| **Distance (Å)** | | |
| --- | --- | --- |
| Bond | Experimental | Theoretical |
| Ga1–O1 | 1.984(2) | 1.942 |
| Ga1–O3 | 1.970(3) | 1.965 |
| Ga1–O5 | 1.939(2) | 1.947 |
| Ga1–O5^i^ | 1.949(2) | 1.962 |
| Ga1–N1 | 2.096(3) | 2.085 |
| R | **0.940** | |
| **Angle (°)** | | |
| Angle | Experimental | Theoretical |
| O1–Ga1–O3 | 91.87(11) | 103.48 |
| O1–Ga1–O5 | 92.21(10) | 98.24 |
| O1–Ga1–O5^i^ | 170.00(10) | 166.66 |
| O1–Ga1–N1 | 82.27(11) | 83.28 |
| O1–Ga1–N2 | 92.75(11) | 86.70 |
| O3–Ga1–O5 | 90.65(11) | 89.45 |
| O3–Ga1–O5^i^ | 94.93(10) | 89.84 |
| O3–Ga1–N1 | 171.12(11) | 172.52 |
| O3–Ga1–N2 | 82.48(11) | 81.17 |
| O5–Ga1–O5^i^ | 80.42(11) | 80.97 |
| O5–Ga1–N1 | 96.22(11) | 92.75 |
| O5–Ga1–N2 | 171.65(11) | 170.22 |
| O5^i^ –Ga1–N1 | 91.77(11) | 83.45 |
| O5^i^ –Ga1–N2 | 95.39(11) | 96.16 |
| N1–Ga1–N2 | 91.09(12) | 96.21 |
| R | **0.989** | |
| Symmetry code: (i) 1 – *x*, –*y*, 2 – *z* | | |

Table S3: Hydrogen bonding interactions in complex **1**.

| **D–H···A** | **D–H (Å)** | **H···A (Å)** | **D···A (Å)** | **D–H···A (^o^)** |
| --- | --- | --- | --- | --- |
| N1–H1···O2^ii^ | 0.97(2) | 2.59(3) | 3.233(4) | 124(3) |
| N1–H1···O3^i^ | 0.97(2) | 2.48(3) | 3.289(4) | 141(3) |
| N2–H2···O4^iii^ | 0.97(3) | 1.97(3) | 2.898(4) | 161(3) |
| O5–H5···O2^ii^ | 0.83(3) | 1.93(3) | 2.740(3) | 166(4) |
| O6–H6C···O4^iii^ | 0.86(5) | 2.02(5) | 2.864(5) | 166(6) |
| O6–H6D···O1^iv^ | 0.86(5) | 2.46(5) | 3.293(5) | 162(6) |
| O6–H6D···O2^iv^ | 0.86(5) | 2.40(4) | 3.098(5) | 138(5) |
| C2–H2B···O6^v^ | 0.99 | 2.51 | 3.293(6) | 136 |
| C4–H4A···O3^vi^ | 0.99 | 2.51 | 3.358(5) | 143 |
| C6–H6A···O1^iv^ | 0.99 | 2.57 | 3.390(4) | 140 |

Symmetry codes: (i) 1 – *x*, –*y*, 2 – *z*; (ii) 1 − *x*, 1 – *y*, 2 – *z*; (iii) 1 − *x*, ½ + *y*, 3/2 – *z*; (iv) 1 − *x*, –½ + *y*, 3/2 – *z*; (v) –*x*, ½ + *y*, 3/2 – *z*; (vi) –1 + *x*, *y*, *z*.

Table S4: Cremer and Pople puckering parameters [69] of the five- and six-membered chelate rings in uns*-cis*-[Ga(1,3-pdda)(*µ*-OH)]_2_^.^2H_2_O (**1**) and uns*-cis*-[Co(1,3-pdda)(*µ*-H_2_O)]_2_^.^4H_2_O complexes.

| Complex | M–N–C–C–C–N | M–N–C–C–O (G) | M–N–C–C–O (R) |
| --- | --- | --- | --- |
| [Ga(1,3-pdda)(*µ*-OH)]_2_^.^2H_2_O (**1**) | Q = 0.604 Å; θ_2_ = 5.1°; ϕ_2_ =189.4°;  q_2_ = 0.054 Å; q_3_ = 0.602 Å  *chair* | ϕ_2_ = 146.7°;  q_2_ = 0.214 Å  *envelope* | ϕ_2_ = 128.8°;  q_2_ = 0.083 Å  *twist* |
| [Co(1,3-pdda)(*µ*-H_2_O)]_2_^.^4H_2_O [70] | Q = 0.605 Å; θ_2_ = 2.7°; ϕ_2_ = 96.0°;  q_2_ = 0.029 Å; q_3_ = 0.604 Å  *chair* | ϕ_2_ = 311.0°;  q_2_ = 0.110 Å  *twist* | ϕ_2_ = 284.8°;  q_2_ = 0.126 Å  *envelope* |

[69] D. Cremer and J. A. Pople, “A general definition of ring puckering coordinates,” *Journal of the American Chemical Society,* vol. 97, no. 6, pp. 1354–1358, 1975*.*

[70] H-H. Chen, D-C. Xia and J-F. Ma, “Crystal structure of aqua[1,3-propanediaminediacetato]-cobalt(II) dihydrate, Co(H_2_O)(C_7_H_12_N_2_O_4_)·2H_2_O,” *Zeitschrift fur Kristallographie* – *New Crystal Structures*, vol. 224, no. 4, pp. 623–624, 2009.

Table S5: Analysis of octahedral distortion in dinuclear six-coordinate 1,3-pdda^2−^ complexes of gallium(III) and cobalt(II) of uns*-cis* configuration.

|  | [Ga(1,3-pdda)(*µ*-OH)]_2_^.^2H_2_O (**1**) | [Co(1,3-pdda)(*µ*-H_2_O)]_2_^.^4H_2_O [70] |
| --- | --- | --- |
| ∑Δ(*O_h_*)*^a^* | 52° | 35° |
| Δ∑(T)*^b^* | +27° | +27° |
| Δ∑(R)*^c^* | +1° | 0° |
| Δ∑(G)*^d^* | –2° | 0° |
| Δ(M–O–C)*^e^* (R) | +8° | +7 |
| Δ(M–O–C)*^e^* (G) | +8° | +6 |
| C_T_–N_T,R_–M | 115.8° Δ = +6.3° | 116.9° Δ = + 7.4° |
| C_R_–N_T,R_–M | 109.3° Δ = −0.2° | 109.0° Δ = −0.5° |
| C_T_–N_T,R_– C_R_ | 112.5° Δ = + 3.0° | 113.7° Δ = +4.2° |
| C_T_–N_T,G_–M | 117.6° Δ = +8.1° | 111.0° Δ = +1.5° |
| C_G_–N_T,G_–M | 108.5° Δ = −1.0° | 109.5° Δ = 0.0° |
| C_T_–N_T,G_–C_G_ | 110.9° Δ = +1.4° | 111.0° Δ = +1.5° |

*^a^*ΣΔ(*O*_h_) is the sum of the absolute values of the deviations from 90° of the octahedral bond angles around the central metal ion. All values rounded off to the nearest degree; *^b^*ΔΣ(T) is the deviation of the 1,3-propanediamine rings’ bond angle sum from the ideal 637.5°; *^c^*Δ∑(R) is the deviation from the ideal (538.5°) of the axial (or out-of-plane) glycinate rings’ bond angle sum. *^d^*Δ∑(G) is the deviation from the ideal (538.5°) of the equatorial (or in-plane) glycinate rings’ bond angle sum; *^e^*Δ(M–O–C) (ring) is the deviation of the corresponding rings’ M–O–C bond angle from 109.5°. Theoretically, this angle can vary from 109.5 to 120° depending on the degree of covalency of the M–O bond.

[70] H-H. Chen, D-C. Xia and J-F. Ma, “Crystal structure of aqua[1,3-propanediaminediacetato]-cobalt(II) dihydrate, Co(H_2_O)(C_7_H_12_N_2_O_4_)·2H_2_O,” *Zeitschrift fur Kristallographie* – *New Crystal Structures*, vol. 224, no. 4, pp. 623–624, 2009.

Table S6: The IR positions of asymmetric (*ν*_asym_) and symmetric (*ν*_sym_) stretching frequencies (cm^-1^) of carboxyl groups of 1,3-H_2_pdda^.^2HCl in comparison to those for the coordinated carboxylate groups in complexes **1** – **3**.

| Compound | *ν*_asym_ | *ν*_sym_ |
| --- | --- | --- |
| 1,3-H_2_pdda^.^2HCl | 1746*vs* | 1430*m*; 1417*m*; 1403*m* |
| uns*-cis-*[Ga(1,3-pdda)(*µ*-OH)]_2_^.^2H_2_O (**1**) | 1656*s*; 1623*vs* | 1394*m*; 1384*m*; 1376*m*; 1366*m* |
| Na[Ga(1,3-pdta)] ^.^3H_2_O (**2**) [34] | 1678*vs*; 1646*vs*; 1626*vs* | 1370*vs*; 1335*vs* |
| Ba[Ga(1,3-pndta)]_2_^.^3H_2_O (**3**) [34] | 1639*vs* | 1370*s* |

*vs* = very strong, *s* = strong, *m* = medium

[34] B. V. Pantović, D. P. Ašanin, F. Perdih, D. D. Radanović, I. Turel, M. I. Djuran, B. Đ. Glišić, “Gallium(III) complexes with 1,3-pdta-type of ligands: the influence of an alkyl substituent in 1,3-propanediamine chain and the metal counter cation on the structural properties of the complex,” *Polyhedron*, vol. 258, p. 117045, 2024.

Table S7: The experimental and calculated chemical shifts (*δ*, ppm) in the ^1^H and ^13^C NMR spectra of complex **1**.

| Atoms | ^1^H NMR | | ^13^C NMR | |
| --- | --- | --- | --- | --- |
|  | Experimental | Theoretical | Experimental | Theoretical |
| C2 (H2) | 1.85 | 1.75 | 22.46 | 21.15 |
| C1/C3 (H1/H3) | 3.12 | 3.22 | 44.23 | 49.29 |
| C4 (H4) | 3.69 | 3.55 | 48.64 | 48.20 |
| C5 | – | – | 170.31 | 174.31 |
| MAE | 0.11 | | 5.13 | |
| R | 0.993 | | 0.999 | |


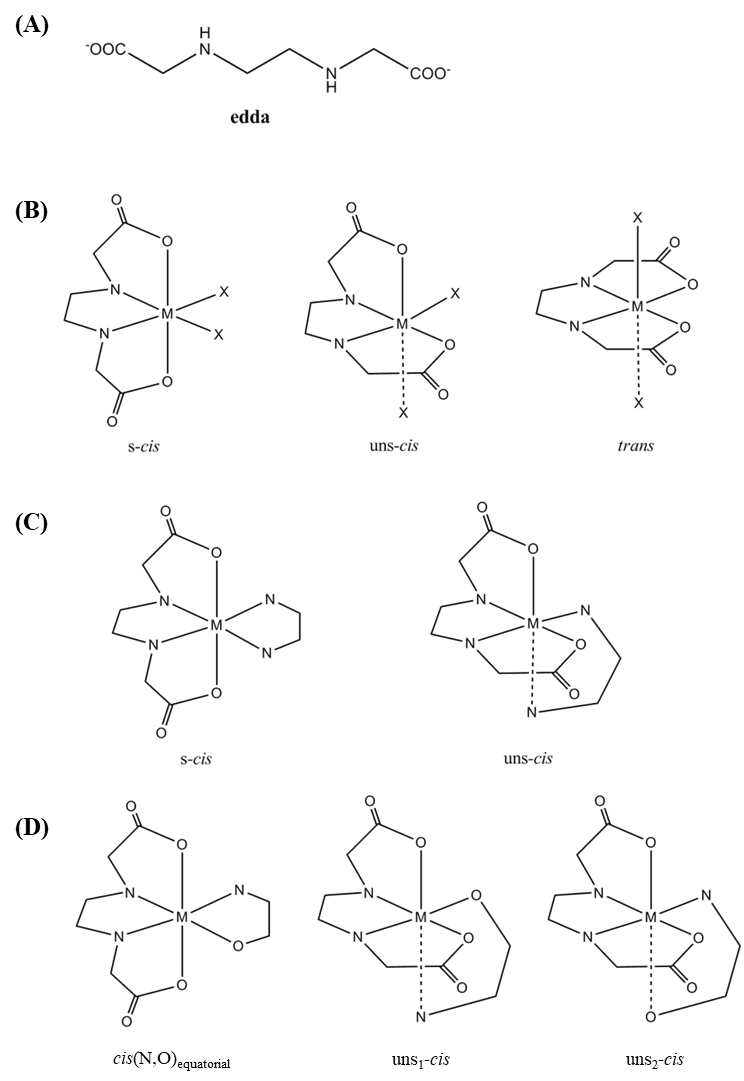


Figure S1: The structural formula of ethylenediamine-*N,N’*-diacetate (edda^2˗^) ligand (A) and possible geometrical isomers for the octahedral metal complexes containing tetradentate edda^2-^ and two monodentate (X) ligands (B).


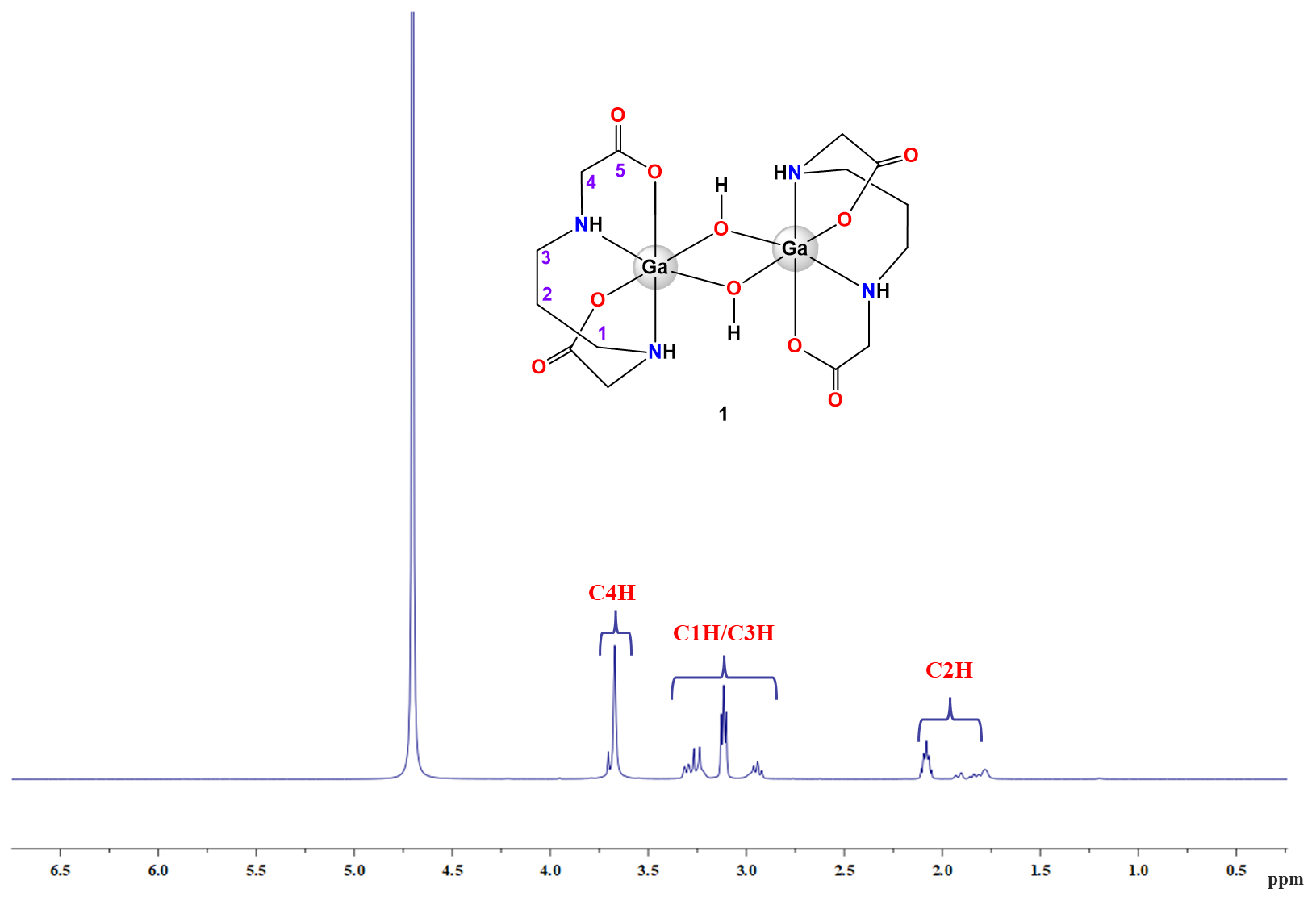


Figure S2: ^1^H NMR spectrum of complex **1** in D_2_O at 600 MHz at room temperature.

Figure S3: ^13^C NMR spectrum of complex **1** in D_2_O at 150 MHz at room temperature.


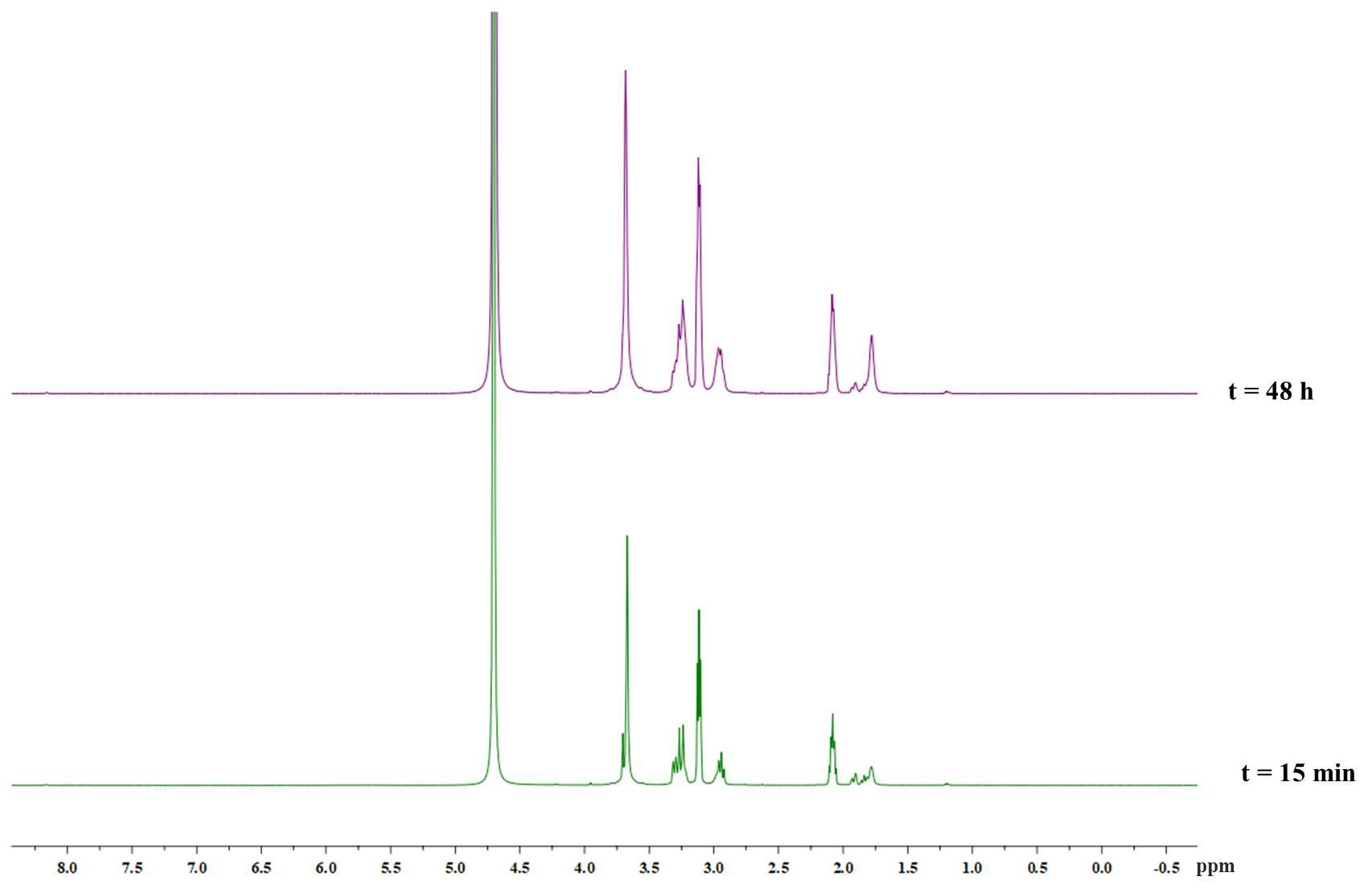


Figure S4: ^1^H NMR spectra of complex **1** recorded immediately and 48 h after dissolution in D_2_O at room temperature (600 MHz).


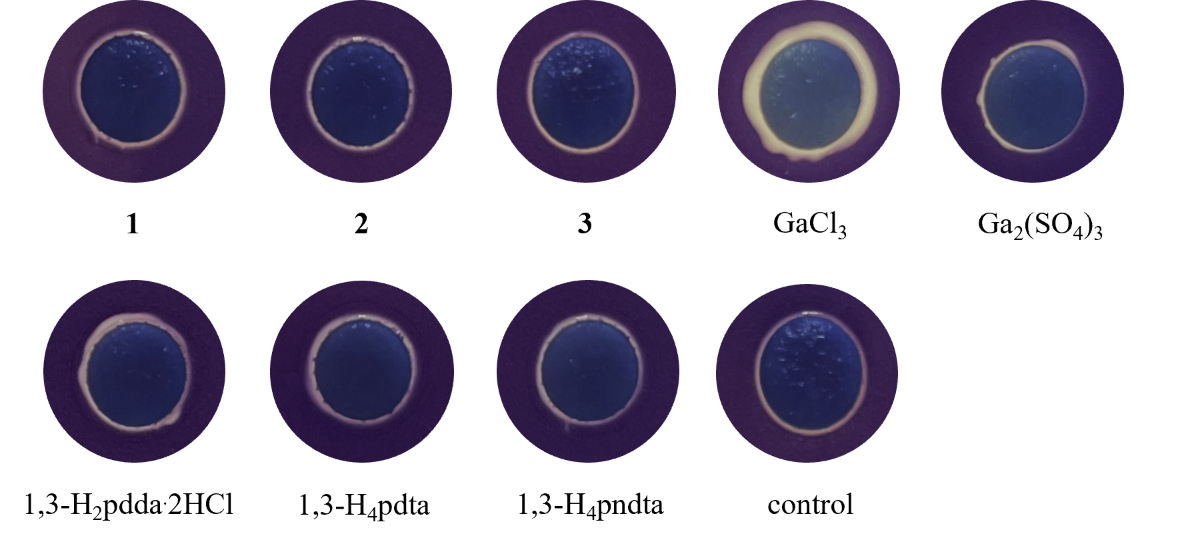


Figure S5: Inhibition of violacein production in the presence of complexes **1** – **3**, salts used for their synthesis (GaCl_3_ and Ga_2_(SO_4_)_3_) and corresponding aminocarboxylic acids on *Chromobacterium* *violaceum* CV026 at 100 µg/disc. H_2_O was used as a control.


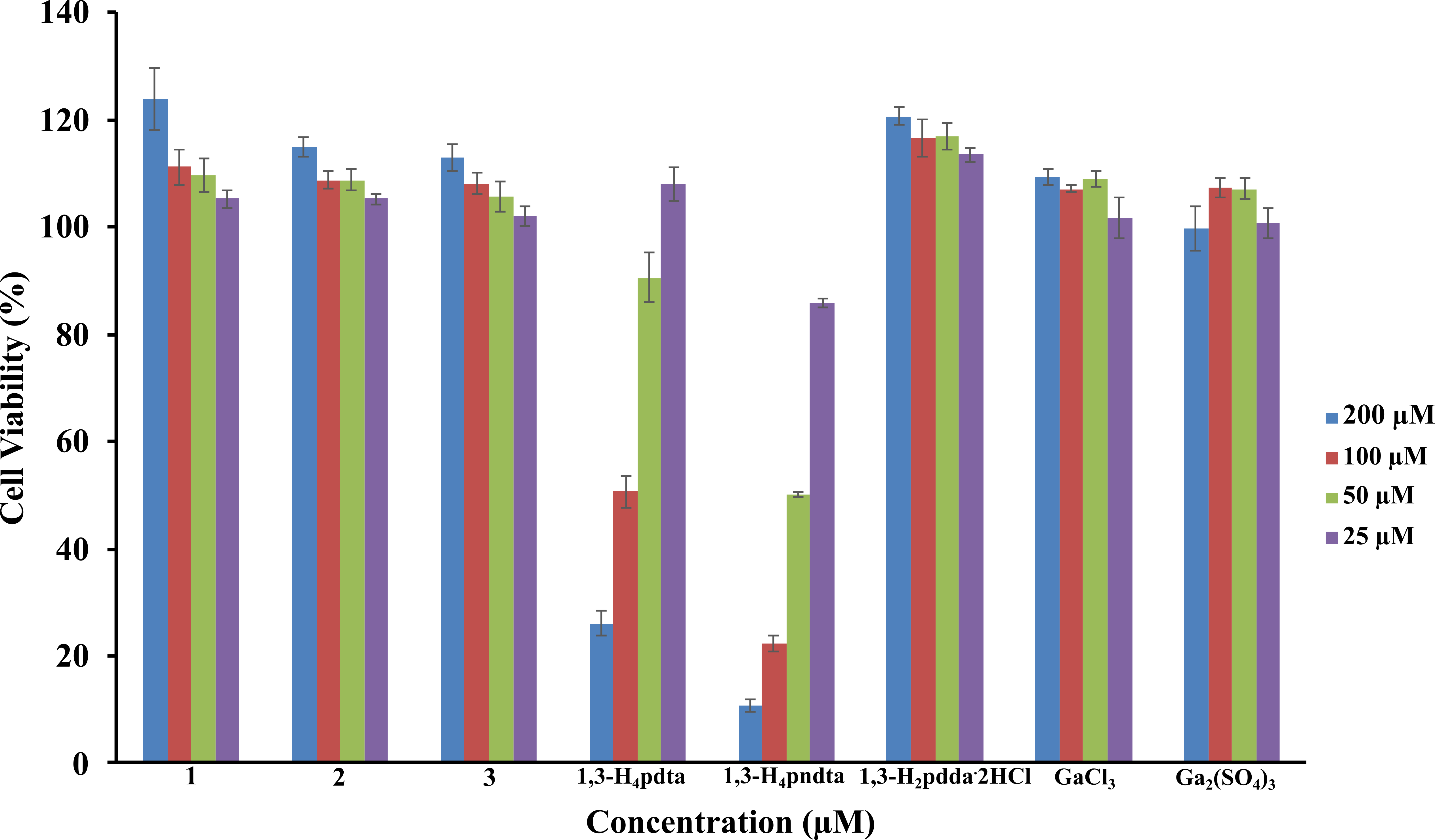


Figure S6: Cytotoxic effect of complexes **1** – **3**, corresponding aminocarboxylic acids and gallium(III) salts on the MRC-5 cells. Values are presented as mean ± SD.


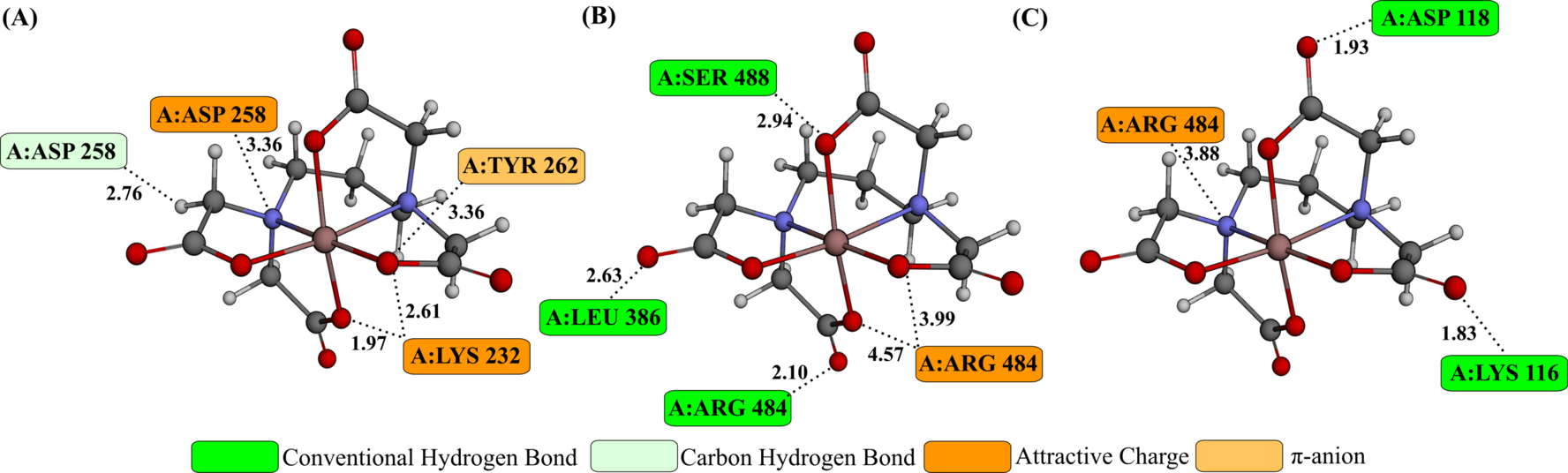


Figure S7: 2D representation of the interactions between complex **2** and BSA with interatomic distances (Å) obtained from the molecular docking study in different active sites: I (A), II (B), and III (C). Different colours indicate the types of interactions (see legend). Legend: Ga – pink, C – grey, H – white, O – red, N – blue.


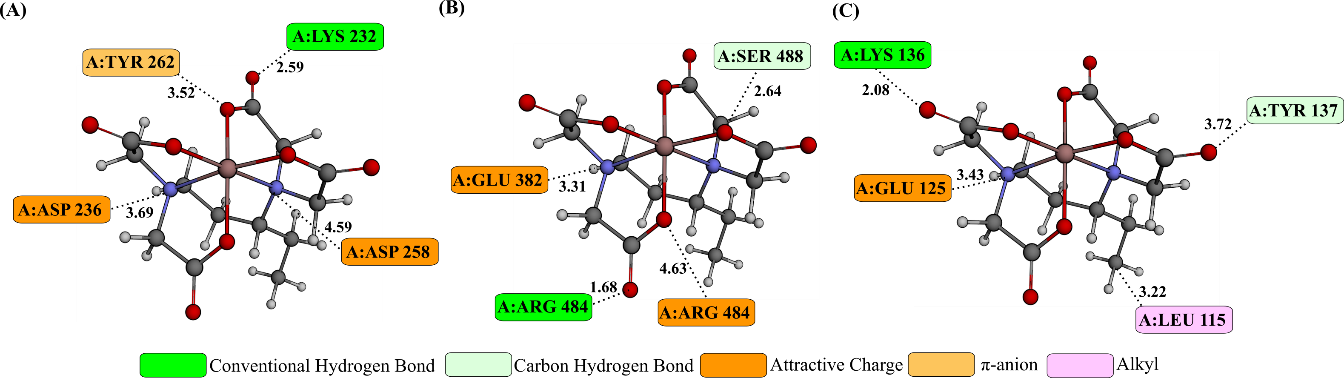


Figure S8: 2D representation of the interactions between complex **3** and BSA with interatomic distances (Å) obtained from the molecular docking study in different active sites: I (A), II (B), and III (C). Different colours indicate the types of interactions (see legend). Legend: Ga – pink, C – grey, H – white, O – red, N – blue.


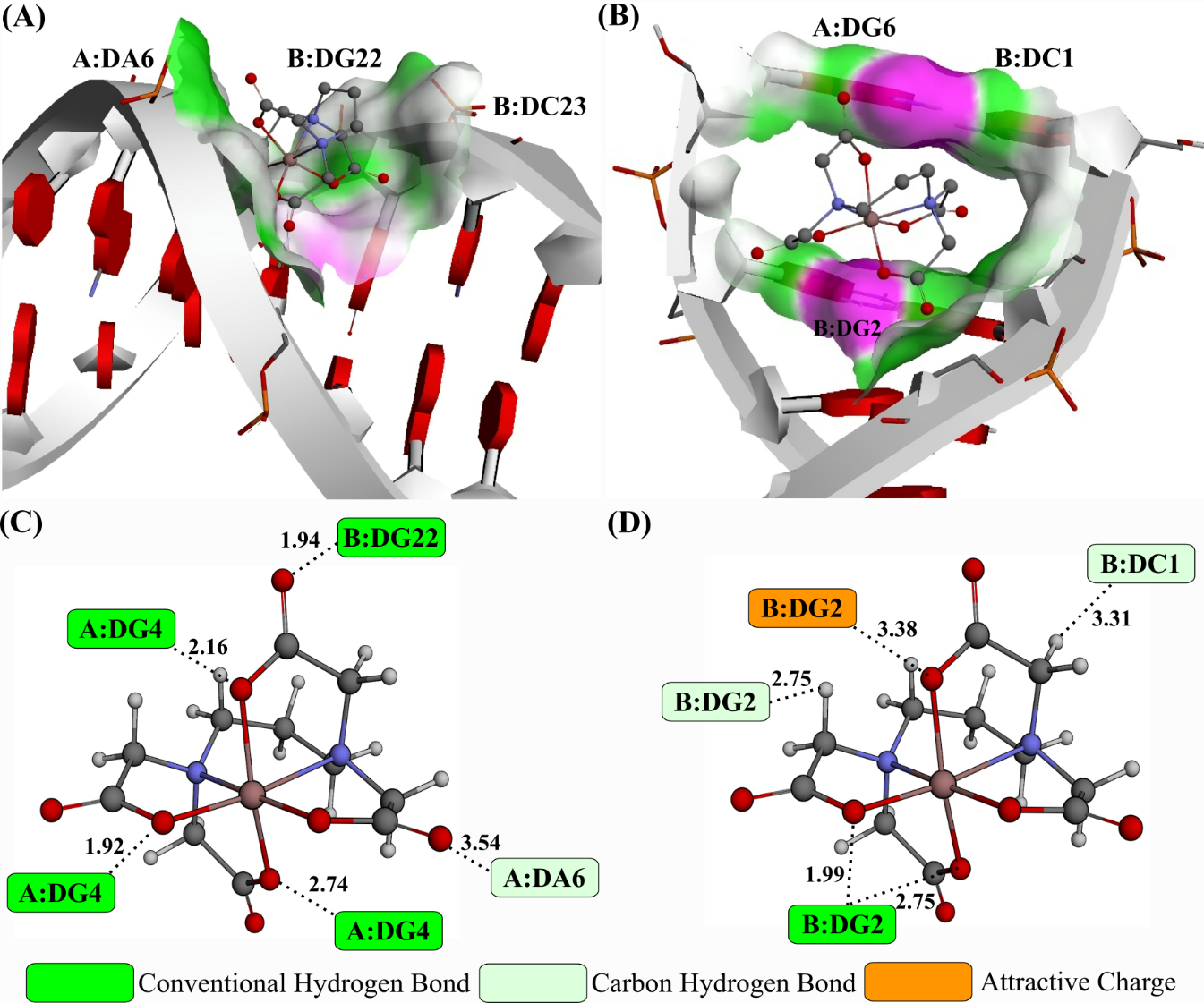


Figure S9: Three-dimensional representations show the most stable conformations of complex **2** as intercalator in (A) 10-bp-DNA (PDB: **1BNA**) and minor groove binder in (B) 6-bp-DNA (PDB: **1Z3F**), with the sugar-phosphate backbones depicted as helically twisted white bands and nucleobases in blue. Figures (C) and (B) highlight the interactions between complex **2** and both DNA sequences, showing interatomic distances (Å) from docking simulations. Nucleotides are labelled (DA = deoxyadenosine; DG = deoxyguanosine; DC = deoxycytidine; DT = deoxythymidine), with interaction types colour-coded. The investigated compounds are shown as grey carbon sticks, and atoms are represented by spheres: N (blue), O (red), H (white) and Ga (pink).


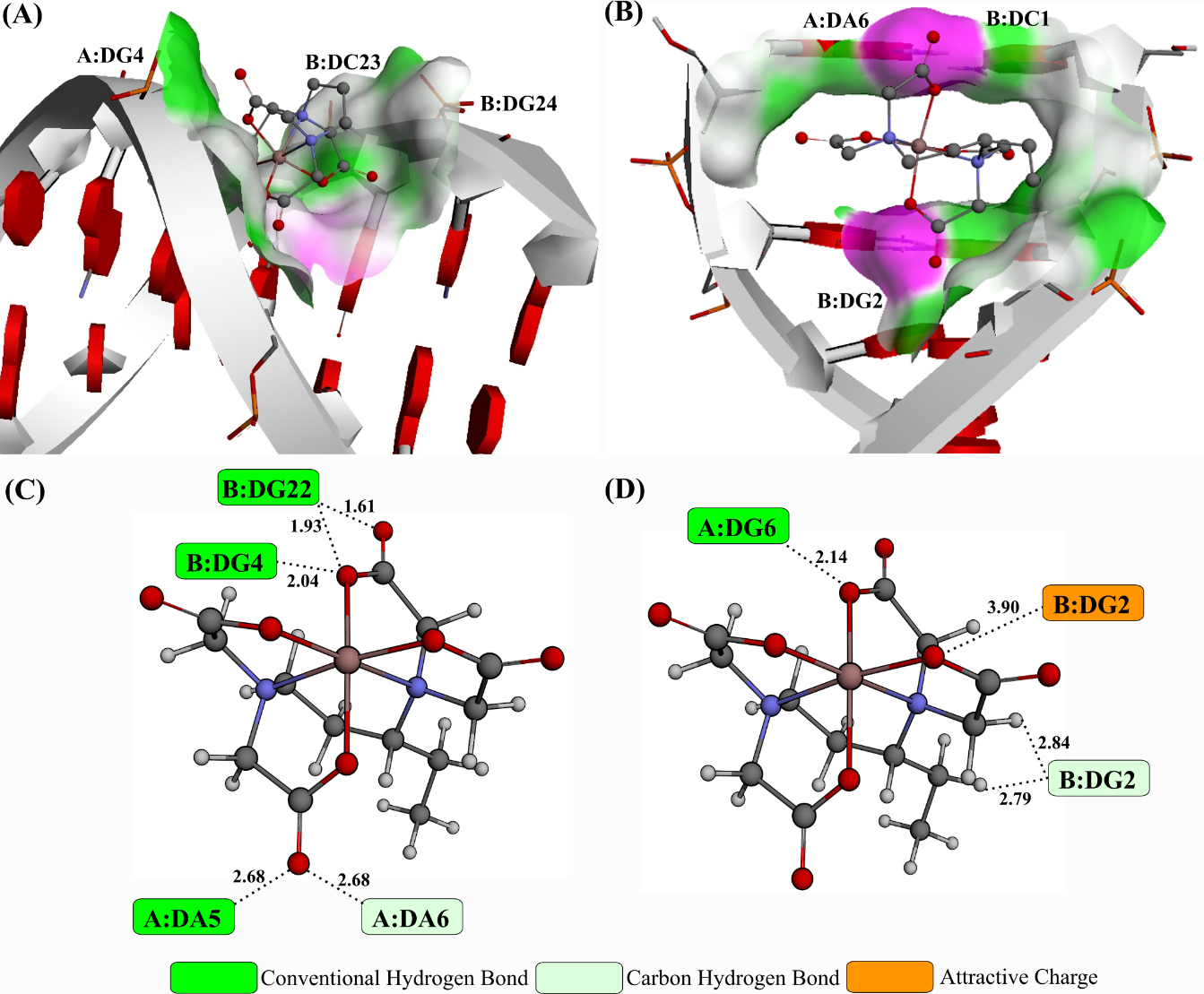


Figure S10: Three-dimensional representations show the most stable conformations of complex **3** as intercalator in (A) 10-bp-DNA (PDB: **1BNA**) and minor groove binder in (B) 6-bp-DNA (PDB: **1Z3F**), with the sugar-phosphate backbones depicted as helically twisted white bands and nucleobases in blue. Figures (C) and (B) highlight the interactions between complex **3** and both DNA sequences, showing interatomic distances (Å) from docking simulations. Nucleotides are labelled (DA = deoxyadenosine; DG = deoxyguanosine; DC = deoxycytidine; DT = deoxythymidine), with interaction types colour-coded. The investigated compounds are shown as grey carbon sticks, and atoms are represented by spheres: N (blue), O (red), H (white) and Ga (pink).
